# Induction of Broadly Neutralizing HIV Antibodies by a Two-Step Mechanism Informs Vaccine Design

**DOI:** 10.1126/science.aec6396

**Published:** 2026-06-18

**Authors:** Ashwin N. Skelly, Harry B. Gristick, Hui Li, Edem Gavor, Andrew J. Connell, Edward F. Kreider, Lorie Marchitto, Michael P. Hogarty, Maddy L. Newby, Joel D. Allen, Weimin Liu, Anthony P. West, Kasirajan Ayyanathan, Mary S. Campion, Kaitlyn Winters, Colette G. Gordon, Rebecca A. Osbaldeston, Macy J. Akeley, Emily Lewis, Yingying Li, Ajay Singh, Kendra Cruickshank, Younghoon Park, Chengyan Zhao, Xuduo Li, Khaled Amereh, Elizabeth Van Itallie, John W. Carey, Amie Albertus, Andrew T. DeLaitsch, Jennifer R. Keeffe, Melinda G. Lituchy, Agnes A. Walsh, Daniel J. Morris, Rumi Habib, Frederic Bibollet-Ruche, Nitesh Mishra, Gabriel Avillion, Nicholas S. Koranda, Samantha J. Plante, Christian L. Martella, Jinery Lora, Eric J. D. Wang, Mark G. Lewis, Malcolm A. Martin, Michel C. Nussenzweig, Michael S. Seaman, Darrell J. Irvine, Kevin J. Wiehe, Barton F. Haynes, Kshitij Wagh, Bette Korber, Raiees Andrabi, Max Crispin, Drew Weissman, Pamela J. Bjorkman, Beatrice H. Hahn, George M. Shaw

**Affiliations:** 1 Departments of Medicine and Microbiology, Perelman School of Medicine, University of Pennsylvania; Philadelphia, PA 19104, USA.; 2 Division of Biology and Biological Engineering, California Institute of Technology; Pasadena, CA 91125, USA.; 3 Penn Institute for RNA Innovation, Perelman School of Medicine, University of Pennsylvania, Philadelphia; PA 19104, USA.; 4 School of Biological Sciences, University of Southampton; Southampton SO17 1BJ, UK.; 5 Duke Human Vaccine Institute and Department of Medicine, Duke University School of Medicine; Durham, NC 27710, USA.; 6 Department of Immunology and Microbiology, Scripps Research Institute; La Jolla, CA 92037, USA.; 7 Bioqual, Inc.; Rockville, MD 20850, USA.; 8 Laboratory of Molecular Microbiology, National Institute of Allergy and Infectious Diseases, National Institutes of Health; Bethesda, MD 20892, USA.; 9 Laboratory of Molecular Immunology, Rockefeller University; New York, NY 10065, USA.; 10 Howard Hughes Medical Institute, Rockefeller University; New York, NY 10065, USA.; 11 Center for Virology and Vaccine Research, Beth Israel Deaconess Medical Center; Boston, MA 02215, USA.; 12 Howard Hughes Medical Institute; Chevy Chase, MD, USA.; 13 Los Alamos National Laboratory; Los Alamos, NM 87545, USA.; 14 New Mexico Consortium; Los Alamos, NM 87545, USA.

## Abstract

A major obstacle confronting HIV-1 vaccine and cure research is the lack of an outbred animal model for rapid and consistent induction of broadly neutralizing antibodies (bNAbs). We designed an epitope-focused simian-human immunodeficiency virus (SHIV.5MUT) that elicited broad and potent V3-glycan-targeted antibodies within a year of infection in 14 of 22 macaques compared with 0 of 14 control animals. SHIV.5MUT elicited bNAbs by a two-step mechanism, inducing an initial wave of V1-directed antibodies that selected for Envs with shortened, hypoglycosylated V1 loops, which in turn primed V3-glycan bNAb precursors. Rhesus bNAbs were immunogenetically and structurally diverse, closely resembling human V3-glycan bNAbs. Env-bNAb coevolution revealed a diverse repertoire of bNAb precursors and the Env variants that matured them, yielding a molecular blueprint for vaccine design.

Prophylactic administration of HIV-1 broadly neutralizing antibodies (bNAbs) to naïve rhesus macaques reliably protects them from simian-human immunodeficiency virus (SHIV) challenge (1–3). In humans, the bNAb VRC01 protects against infection by sensitive viral strains (4, 5). When given therapeutically, bNAbs reduce plasma virus load and delay viral rebound after antiretroviral treatment interruption, in rare instances leading to a “functional cure” (6–10). These findings have led to intense efforts to elicit bNAbs by vaccination. While there has been progress, consistent induction of bNAbs in the plasma at titers that approach clinically protective thresholds has not been achieved. A major obstacle has been the lack of an outbred animal model wherein potent bNAbs can be reproducibly elicited and their developmental pathways elucidated. Such a model, if available, could serve as both a blueprint and a benchmark for iterative vaccine design.

The V3-glycan patch on the gp120 subunit of the HIV-1 envelope (Env) glycoprotein is a frequently targeted bNAb site in chronic human infection (11). This epitope includes the N332_gp120_ and N301_gp120_ glycans and the ^324^GDIR_327_ peptide motif at the base of the V3 loop, which contributes to coreceptor binding (12–15). V3-glycan antibodies are among the most potent bNAb classes (16, 17), with the broadest members neutralizing up to 65% of circulating HIV strains (14, 16–21). Unlike bNAbs targeting other epitopes, V3-glycan bNAbs are structurally and immunogenetically diverse (15–17). As such, they arise from a less restricted pool of germline precursors and may be easier to elicit by vaccination. Additionally, compared to other bNAb classes, V3-glycan bNAbs tend to require less somatic hypermutation to acquire neutralization breadth (14, 17, 18). These features make V3-glycan bNAbs a promising vaccine target.

The most common strategy for eliciting V3-glycan bNAbs has been to use reverse vaccinology approaches, in which the germline precursor of a mature bNAb is inferred and used as a template to design immunogens that selectively bind to it (17, 22). However, such “lineage-based” strategies are limited by a paucity of high-confidence inferences of V3-glycan bNAb unmutated common ancestors (UCAs). Inferred germlines (iGLs) have been used in their stead, but these typically retain most of their mature heavy chain complementarity-determining region 3 (CDRH3) sequence. This is particularly problematic when targeting V3-glycan bNAb lineages, which generally bind in a CDRH3-dominant manner (12–15). Additionally, engineering an immunogen to maximize affinity for a single UCA/iGL does not ensure cross-reactivity with other bNAb precursors targeting the same epitope. Despite these limitations, advances have been made using immunogens designed to engage precursors of the human V3-glycan bNAbs PGT121 (23–26), DH270 (27, 28), and BG18 (29–31). Sequential immunization in immunoglobulin knock-in mouse models provided proof-of-principle that these types of lineages can be primed and boosted *in vivo* (24, 27, 31), although consistent V3-glycan bNAb induction resulting in protective plasma titers has not yet been achieved in outbred animals (26, 30).

As an alternative to lineage-based design, we describe here a lineage-agnostic, epitope-focused approach to eliciting V3-glycan bNAbs. Previously, we hypothesized that immunizing with BG505.N332 Env variants lacking N-glycans at gp120 residues N133/N137/N156 (RC1) (25) or N133/N137 (11MUTB) (23) might selectively prime V3-glycan bNAb precursors, and that further boosting with more native-like immunogens including 5MUT (23) would promote their maturation to breadth (26). This approach was only partially successful, eliciting antibodies that exhibited weak cross-neutralization and bound open or occluded-open Env conformations (26, 32, 33). Additionally, structural analyses suggested that while the initial humoral response was focused on the V3 region, it became increasingly off-target with subsequent boosting (26). In the current study, we substituted the boosting immunogen (5MUT) with an infectious SHIV bearing the corresponding Env. We reasoned that infection with SHIV.5MUT might better mature RC1/11MUTB-primed responses by acting as an “evolving immunogen” given its persistent replication, high antigenic load, and ability to coevolve with the humoral response.

## Results

### Consistent induction of plasma breadth in SHIV.5MUT-infected macaques

**Fig. 1.**
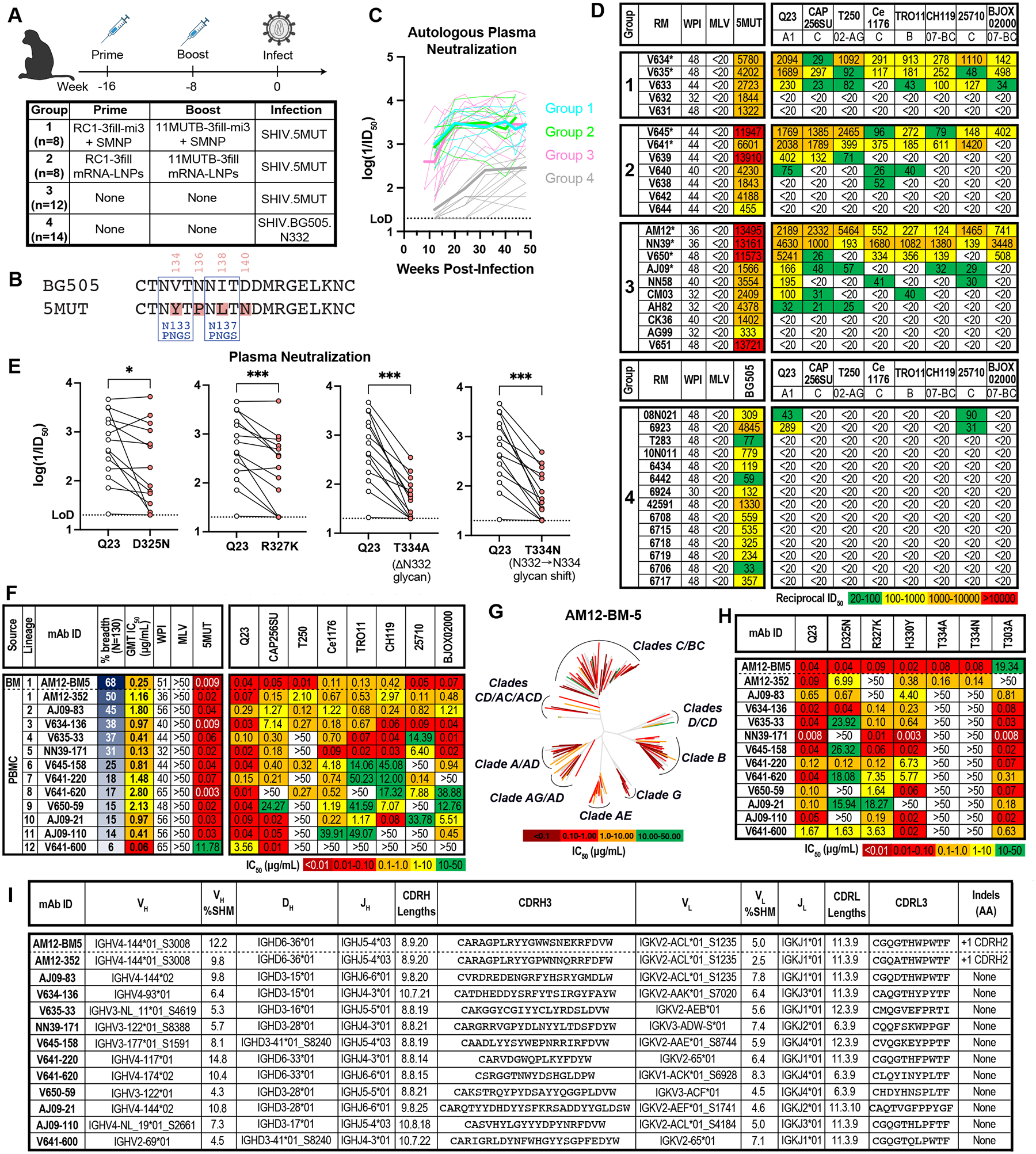
SHIV.5MUT rapidly and consistently induces V3-glycan bNAbs. **(A)** Study design. Macaques were immunized with protein nanoparticles adjuvanted with SMNP or mRNA-LNPs at eight-week intervals. All vaccinated as well as unvaccinated control animals were then infected with SHIV.5MUT or SHIV.BG505.N332. **(B)** Amino acid alignment of BG505.N332 and 5MUT Envs in the V1 region. Differences at positions 134, 136, 138, and 140 are highlighted in red, and PNGSs are boxed. **(C)** Autologous plasma neutralization titers are shown for SHIV.5MUT- (Groups 1–3) and SHIV.BG505.N332- (Group 4) infected macaques, represented as log(1/ID_50_). Thin lines represent individual animals and thick lines represent group mean. **(D)** Plasma neutralization titers (1/ID_50_) are shown for each animal against eight heterologous viruses at the timepoint of greatest breadth within 48 weeks post-infection (WPI). Asterisks indicate animals from which bNAbs were isolated. **(E)** The heterologous neutralizing response of 14 SHIV.5MUT-infected macaques was mapped against Q23 mutants lacking key V3-glycan epitope residues, with titers expressed as log(1/ID_50_). Each point represents a single macaque with lines connecting paired samples. Wilcoxon matched-pairs signed-rank test was used. *P < 0.05; ***P < 0.001. **(F)** Neutralization profiles of monoclonal antibodies representing the twelve V3-glycan bNAb lineages isolated from eight SHIV.5MUT-infected macaques. Titers are expressed as IC_50_ in μg/mL. **(G)** Neutralization breadth and potency of the AM12-BM5 bNAb against a 130-strain global virus panel, with a dendrogram illustrating the phylogenetic relatedness of tested Envs. Neutralization potency is indicated with a heatmap. **(H)** Epitope mapping of rhesus bNAbs using a panel of Q23 mutant viruses lacking key V3-glycan epitope residues. Titers are expressed as IC_50_ in μg/mL. **(I)** Immunogenetic features of rhesus V3-glycan bNAbs. Somatic hypermutation (SHM) is calculated at the nucleotide level and complementarity-determining region (CDR) lengths are expressed as amino acids. SMNP, saponin/MPLA nanoparticle; LNP, lipid nanoparticle; PNGS, potential N-linked glycosylation site; MLV, murine leukemia virus; RM, rhesus macaque; BM, bone marrow; PBMC, peripheral blood mononuclear cell.

Macaques were sequentially immunized with RC1 and 11MUTB as either protein nanoparticles (Group 1, n=8) or mRNA-LNPs encoding stabilized, membrane-anchored Env trimers (Group 2, n=8) and then infected with SHIV.5MUT eight weeks later. Two additional groups of unimmunized macaques were infected with either SHIV.5MUT (Group 3, n=12) or wildtype SHIV.BG505.N332 (Group 4, n=14) (Fig. 1A, Data S1). Immunogen design details are in the Supplementary Text (fig. S1A–B). Vaccinated animals developed robust autologous neutralizing plasma responses within eight weeks of the RC1 prime, with higher mean titers in protein- versus mRNA-immunized animals (fig. S1C). Titers increased upon boosting with 11MUTB, with a dose escalation subgroup of protein-vaccinated macaques reaching higher titers than the bolus subgroup (fig. S1C). Following vaccination, there was no cross-neutralization of viruses bearing 5MUT or BG505.N332 Envs, indicating successful immunofocusing of the humoral response to the glycan-depleted V1V3 region of RC1 and 11MUTB (fig. S1C). Alanine scanning in an 11MUTB backbone revealed that the bulk of the neutralizing response targeted residues N137_gp120_ and L139_gp120_ (fig. S1D).

We aimed to mature the vaccine-elicited response by infecting with the “evolving immunogen” SHIV.5MUT (Fig. 1A). The 5MUT and BG505.N332 Envs differ at only four positions: V1 loop residues V134Y, N136P, I138L, and D140N (Fig. 1B). These substitutions were originally selected to enhance binding to a partially germline-reverted form of the bNAb PGT121 (23). SHIV.5MUT exhibited wildtype BG505.N332-like infectivity in TZM-bl cells and a tier-2 neutralization phenotype. Antibodies targeting regions exposed on Envs with more open conformations such as CD4i, V2i, and linear V3 epitopes generally failed to neutralize SHIV.5MUT, while V2-apex bNAbs that recognize quaternary epitopes did so potently (fig. S1E). Additionally, SHIV.5MUT exhibited enhanced susceptibility to multiple V3-glycan bNAbs, confirming that the mutations in 5MUT increased epitope accessibility (23) (fig. S1E).

Animals in Groups 1, 2, and 3 were inoculated with SHIV.5MUT and animals in Group 4 with SHIV.BG505.N332 (Data S1). Productive infection ensued in all 42 macaques (fig. S1F). Five macaques with sustained high viral loads rapidly progressed to AIDS, whereas one macaque controlled infection, exhibiting low-level viremia and no detectable neutralization of the autologous SHIV.5MUT (fig. S1G). These six animals were excluded from further analysis. The remaining 36 macaques were followed for one year, during which no difference in setpoint viral load was observed between SHIV.5MUT- and SHIV.BG505.N332-infected macaques (fig. S1H), indicating that viral load is not a confounding factor.

Within the first 12 weeks of infection, all three groups of SHIV.5MUT-infected macaques developed potent autologous neutralizing responses that increased over time, plateaued by week 20, and persisted until at least week 48 (Fig. 1C). SHIV.BG505.N332-infected animals exhibited similar responses but with lower autologous neutralization titers (Fig. 1C). We observed heterologous neutralization activity in the plasma of SHIV.5MUT-infected macaques as early as week 20 post-infection, regardless of vaccination history (Data S2). Overall, 14 of 22 SHIV.5MUT-infected macaques developed bNAb responses, defined as neutralization of at least 3 of 8 heterologous viruses within 48 weeks of infection. Eight animals neutralized at least 6 of 8 viruses in the panel with 50% inhibitory dose (ID_50_) titers frequently exceeding 1:1000 (average GMT ID_50_ = 1:507) (Fig. 1D). All bNAb responses mapped to the V3-glycan epitope (Fig. 1E). In contrast, 0 of 14 SHIV.BG505.N332-infected macaques developed plasma breadth within the same 48-week timeframe (p<0.0001, Fisher’s exact test) (Fig. 1D, Data S3). The frequency of bNAb elicitation was not different between vaccinated (Groups 1–2) and unvaccinated (Group 3) SHIV.5MUT-infected animals, suggesting that SHIV.5MUT or a derivative induced these bNAb lineages rather than the RC1 or 11MUTB immunogens (Fig. 1D). Both plasma viral load (fig. S2A) and autologous neutralization titers (fig. S2B) were higher in SHIV.5MUT-infected macaques that developed bNAbs compared to those that did not, suggesting that antigen drive contributed to bNAb elicitation.

### Isolation of immunogenetically diverse V3-glycan bNAbs

We isolated heterologous Env-binding IgG^+^ B cells from peripheral blood mononuclear cells (PBMCs) of the eight macaques with maximal plasma breadth and sequenced their antibody genes (fig. S3A). Representatives of expanded lineages were synthesized as recombinant monoclonal antibodies (mAbs) and screened for neutralization activity. In total, we screened 238 mAbs representing 106 distinct lineages (Data S4), yielding twelve V3-glycan bNAb lineages (Fig. 1F). We also sorted bone marrow plasma cells from the macaque with the greatest plasma breadth (fig. S3B) and identified additional bNAb lineage members, one of which (AM12-BM5) potently neutralized all viruses in the screening panel (Fig. 1F).

To characterize the breadth of these bNAbs more comprehensively, we assessed their neutralization activity against a 130-virus panel containing representatives of all major HIV-1 clades. Breadth ranged from 6–68% with GMT IC_50_ of 0.06–2.80 μg/mL (Fig. 1F, Data S5), comparable to prototypical human V3-glycan bNAbs (17, 19, 21, 34). Like human V3-glycan bNAbs, most SHIV.5MUT-elicited bNAbs were restricted in their neutralization breadth to viruses containing an N332_gp120_ glycan. The exceptions were AM12-352 and its bone marrow plasma cell-derived relative AM12-BM5, which additionally neutralized viruses lacking the N332_gp120_ glycan (Data S5), thereby broadening their coverage to include clade AE strains from Asia (Fig. 1G, fig. S4A–B) (20, 35).

To map the SHIV.5MUT-elicited bNAbs, we tested their neutralization activity against Q23 mutants that lacked contact residues in the canonical V3-glycan epitope. Consistent with the 130-virus neutralization profiles (Data S5, Fig. 1G), all bNAbs exhibited strict dependence on the N332_gp120_ glycan except AM12-352 and AM12-BM5, which instead required the N301_gp120_ glycan (Fig. 1H). N301_gp120_-glycan-dependence is desirable because this glycan, unlike the N332_gp120_ glycan, is highly conserved across all HIV-1 subtypes (fig. S4B). Additionally, representative mAbs from all lineages required D325_gp120_, R327_gp120_, or H330_gp120_, although the pattern and extent of dependence varied (Fig. 1H). This diversity was observed both between and within animals. We isolated three different V3-glycan bNAb lineages from each of two macaques (AJ09 and V641), and representative antibodies from these lineages exhibited distinct patterns of dependence on residues D325_gp120_, R327_gp120_, and H330_gp120_ (Fig. 1H). We also observed intra-lineage heterogeneity in epitope recognition, with several members of the AM12-352 lineage depending completely on the N332_gp120_ glycan instead of the N301_gp120_ glycan (fig. S5A).

The SHIV.5MUT-induced V3-glycan bNAbs were immunogenetically diverse, utilizing various V_H_3- and V_H_4-family gene segments (Fig. 1I), which comprise the most common V_H_ alleles in both humans and macaques (36, 37). Thus, unlike for V2-apex and CD4-mimetic bNAbs, there was no requirement for particular immunoglobulin alleles (38–41). The range in CDRH3 length was wide (14–25 amino acids, median = 20) and within the range of typical rhesus and human antibodies (Fig. S5B) (42). V3-glycan bNAb lineages did not exhibit any indels except for the AM12-352 lineage, which acquired multiple independent insertions in CDRH2 (fig. S6). The average frequency of V_H_ somatic mutation was 8.4% at the nucleotide level (Fig. 1I), less than half that of previously described V3-glycan bNAbs (17), implying that once primed, these lineages may not require extensive maturation to achieve breadth and potency.

### Structural features of macaque V3-glycan bNAbs

**Fig. 2.**
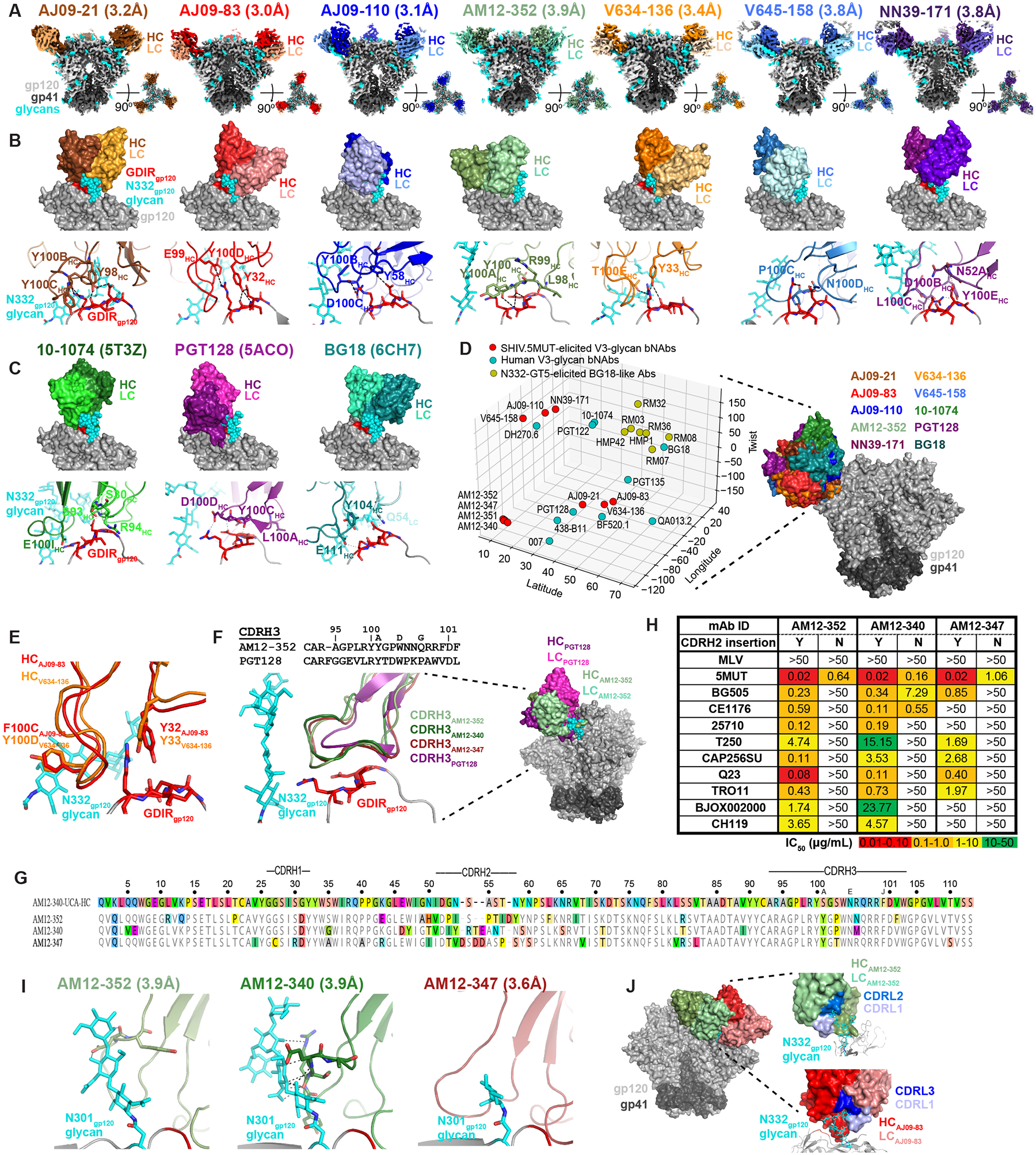
SHIV.5MUT-induced V3-glycan bNAbs are structurally diverse and resemble human bNAbs. **(A)** Cryo-EM maps of SHIV.5MUT-induced V3-glycan bNAbs in complex with 5MUT-3fill. The heavy chain of each bNAb is shown in a darker shade while the light chain is shown in a lighter shade. The N332_gp120_ glycan is modeled as cyan spheres and the ^324^GDIR_327_ peptide motif is red. **(B)** Top, surface representations of the V3-glycan bNAbs from **(A)** bound to gp120, with the N332_gp120_ glycan (cyan) and ^324^GDIR_327_ motif (red). Bottom, interfaces of the predicted interactions between the V3-glycan bNAbs and the ^324^GDIR_327_ motif. **(C)** Top, surface representations of three human-derived V3-glycan bNAbs: 10–1074 (PDB ID: 5T3Z), PGT128 (PDB ID: 5ACO), and BG18 (PDB ID: 6CH7). Bottom, potential interactions between the V3-glycan bNAbs and the ^324^GDIR_327_ motif (red) and N332 glycan (cyan) of gp120. **(D)** Comparative analysis of approach angle between V3-glycan bNAbs elicited by SHIV.5MUT infection (red), V3-glycan bNAbs isolated from humans (blue), and putative V3-glycan bNAb precursors isolated from N332-GT5-immunized macaques (30). Antibodies cluster based on similarity in approach angle. **(E**) Structural overlay of AJ09-83_HC_ and V634-136_HC_ demonstrating shared pairwise interactions with the ^324^GDIR_327_ motif (red) and N332_gp120_ glycan (cyan). **(F)** Amino acid sequence alignment (top left) and structural overlay (bottom left) of the CDRH3 loops from AM12-352 and PGT128. Right, structural overlay of AM12-352 and PGT128 binding to the Env V3-glycan epitope. **(G)** Alignment of the AM12-352 lineage UCA heavy chain amino acid sequence with those of mature bNAbs representing distinct sub-lineages. Differences from the UCA, including indels in CDRH2, are highlighted. **(H)** Influence of CDRH2 insertions on neutralization breadth. The indicated antibodies were produced with (Y) and without (N) their respective CDRH2 insertions. Neutralization activity against a panel of viruses is shown, expressed as IC_50_ in μg/mL. **(I)** Predicted interactions of the CDRH2 loops of AM12-352, AM12-340, and AM12-347 with the N301_gp120_ glycan. **(J)** Left, structural overlay of AM12-352 and AJ09-83. Right, predicted interactions between the CDRL loops of AM12-352 (top) and AJ09-83 (bottom) with the N332_gp120_ glycan. HC, heavy chain; LC, light chain.

We selected ten V3-glycan bNAbs representing seven lineages and determined single-particle cryo-EM structures of their Fab/5MUT Env trimer complexes (Fig. 2A, fig. S7, fig. S8A–D, and Data S6). All ten recognized the V3-glycan epitope, contacting the N332_gp120_ glycan and using heavy chain-encoded tyrosine residues to interact with ^324^GDIR_327_ in a manner similar to human V3-glycan bNAbs (Fig. 2B–C, fig. S8C) (43). Human V3-glycan bNAbs often use a negatively charged residue within CDHR3 (e.g., Glu100I_HC_ in 10–1074) to contact the conserved R327_gp120_ residue (Fig. 2C) (43). This interaction was recapitulated by SHIV.5MUT-elicited bNAbs AJ09-83 and AJ09-110 (Fig. 2B).

We calculated approach angles for the Fab/Env structures and compared them to those of human V3-glycan bNAbs as well as macaque V3-directed antibodies elicited by N332-GT5, a germline-targeting immunogen designed to stimulate BG18-like precursors (Fig. 2D, fig. S9A–B) (30). We found that SHIV.5MUT-elicited bNAbs approached the epitope from a wide range of angles, much like human V3-glycan bNAbs. In contrast, antibodies elicited by N332-GT5 exhibited a narrow range of approach angles that resembled that of BG18, consistent with their restricted immunogenetics (30).

We also assessed whether any of the SHIV.5MUT-elicited bNAb lineages shared a common binding mode, as found for VRC01-class bNAbs (44). Only AJ09-83 and V634-136 shared pairwise Env-antibody interactions (Fig. 2E). These included Phe100C_HC.AJ09-83_ / Tyr100D_HC.V634-136_, which interact with the N-acetylglucosamine of the N332_gp120_ glycan, and Tyr32_HC.AJ09-83_ / Tyr33_HC.V634-136_, which interact with the R327_gp120_ sidechain and I326_gp120_ backbone. The Phe100C_HC.AJ09-83_ / Tyr100D_HC.V634-136_ residues are both contributed by the “YYY” motif encoded by the D3-15*01 gene segment in reading frame 2 (Fig. 1I), although the common pairwise interactions occurred at adjacent positions within this motif. The shared tyrosine-mediated interactions at positions 32_HC.AJ09-83_ / 33_HC.V634-136_ are part of CDRH1 loops encoded by distinct V_H_ gene segments.

We found many structural and immunogenetic similarities between rhesus and human bNAbs. Although PGT128 (human) and AM12-352 (macaque) exhibited different angles of approach and had low CDRH3 amino acid sequence identity (28.6%), both included a CDRH3-encoded LRY motif that formed nearly identical architectures and made similar interactions with ^324^GDIR_327_ (Fig. 2F). Additionally, the evolution of the AM12-352 lineage mirrored that of the PGT128 lineage – both split into sub-lineages defined by CDRH2 insertions that contributed to neutralization breadth (45). Isolated members of the AM12-352 lineage segregated into clades characterized by independent insertions in CDRH2 (Fig. 2G, fig. S6). Representatives of four such clades (AM12-BM5, AM12-352, AM12-340, and AM12-347) demonstrated distinct neutralization profiles, covering 68%, 50%, 32%, and 9% of the 130-virus panel (Data S5). AM12-BM5 and AM12-352, which independently acquired an identical CDRH2 insertion (fig. S6), were the only antibodies capable of neutralizing viruses lacking the N332_gp120_ glycan and exhibited a strong functional dependence on the N301_gp120_ glycan (Fig. 1H and Data S5). Removing the CDRH2 insertions from AM12-352, AM12-340, and AM12-347 markedly diminished their neutralization breadth and potency (Fig. 2H), and Fab/Env structures revealed that these insertions formed distinct architectures, each interacting with the N301_gp120_ glycan in a different manner. The CDRH2 of AM12-352 and AM12-340 formed curved loops that stacked against the face of the N301_gp120_ glycan, whereas the AM12-347 CDRH2 formed a straighter loop that packed against the side of the glycan (Fig. 2I). This is reminiscent of the PGT128 lineage, in which a CDRH2 insertion increased contact with the N332_gp120_ glycan and enhanced breadth against viruses containing this glycan, whereas lineage members without the insertion (such as PGT130) better neutralized N334_gp120_-glycan-containing viruses (45). Finally, while SHIV.5MUT-elicited V3-glycan bNAbs used diverse immunoglobulin gene segments, two bNAbs from different macaques, AM12-352 and AJ09-83, had nearly identical light chains (Fig. 1I). Structural overlay revealed that both light chains engaged the N332_gp120_ glycan, although the Fabs recognized opposing sides of the glycan using CDRL1 and either CDRL2 (AM12-352) or CDRL3 (AJ09-83) (Fig. 2J).

### Ontogeny of rhesus V3-glycan bNAbs

**Fig. 3.**
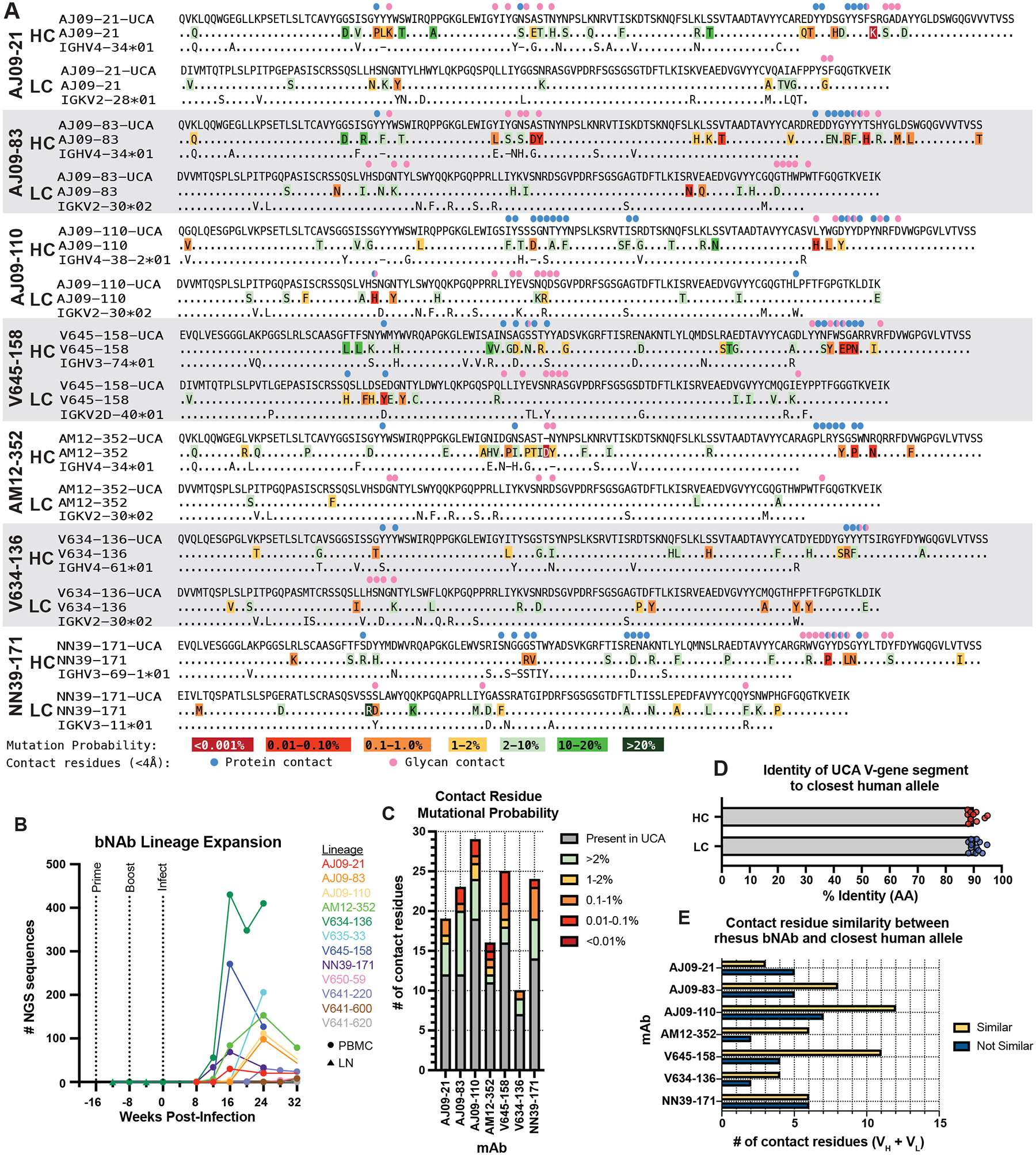
Characterization of inferred V3-glycan bNAb UCAs suggests similar antibodies may be elicitable in humans. **(A)** Alignments of heavy chain (HC) and light chain (LC) amino acid sequences of seven inferred UCAs, the corresponding mature bNAbs, and closest human V_H_/V_K_ alleles are shown. Analysis of the five remaining UCAs is shown in fig. S10. Somatically mutated residues in the mature bNAb sequences are color-coded by estimated mutation probability, as determined by ARMADiLLO (48). Mature bNAb residues that contact amino acids within gp120 (defined as <4Å) are indicated by blue circles, whereas bNAb residues that contact glycans (defined as <4Å) are indicated by pink circles. **(B)** Kinetics of bNAb lineage stimulation and expansion, quantified as the number of sequences from bulk IgG^+^ B cells at each timepoint belonging to the indicated bNAb lineage. Circles indicate sequences were obtained from PBMCs, while triangles indicate sequences were obtained from lymph nodes. **(C)** Number of contact residues encoded by the UCA (grey) compared to those generated by somatic hypermutation for the indicated bNAbs. The somatically mutated contact residues are color-coded as in **(A)**. **(D)** Percent amino acid sequence identity between each UCA V_H_/V_K_ gene segment and the closest human allele. **(E)** Number of contact residues that are biochemically similar in the closest human V_H_ and V_K_ alleles. HC, heavy chain; LC, light chain; NGS, next-generation sequencing; PBMC, peripheral blood mononuclear cell; LN, lymph node; UCA, unmutated common ancestor; AA, amino acid.

Robust inferences of bNAb UCAs and intermediate ancestors define critical stages in bNAb development and can serve as valuable guides for immunogen design (18, 27). An advantage of the SHIV model is that it enables longitudinal B cell receptor (BCR) analyses necessary to infer high-confidence UCAs, which are critical to identifying Env variants capable of priming and boosting bNAb lineages. We sequenced the BCRs of IgG^+^ B cells from draining lymph nodes (post-immunization, Groups 1–2) and PBMCs (post-infection, Groups 1–3) at multiple timepoints as well as naïve IgM^+^IgD^+^ peripheral B cells from one timepoint and used the IgDiscover (46) and SONAR (47) bioinformatic pipelines to infer twelve high-confidence V3-glycan bNAb UCAs from eight macaques (Fig. 3A, fig. S10).

We did not detect bNAb lineage members in the draining lymph node or PBMC compartments of vaccinated animals (Groups 1–2) prior to infection with SHIV.5MUT, suggesting that the immunizations did not prime these bNAb lineages. In contrast, we observed early members within 12–24 weeks of infection in all groups (Fig. 3B), indicating rapid and efficient priming by SHIV.5MUT or a derivative. This timing was consistent with the first detection of plasma neutralization breadth, typically 20–32 weeks post-infection (Data S2).

Given the rapidity with which the bNAb lineages developed breadth (Data S2) and their low rates of somatic mutation (Fig. 1I), we hypothesized that their maturation pathways were relatively simple. Indeed, most structurally identified contact residues were UCA-encoded rather than the product of somatic hypermutation (Fig. 3A, C). Of the non-UCA-encoded contact residues, half were predicted to be “probable” mutations (48), suggesting that they may be relatively easy to induce by vaccination (Fig. 3A, C).

To assess the translational relevance of our findings to humans, we searched the orgdb database (49) for human V_H_ and V_K_ alleles with the highest amino acid identity to our rhesus bNAb UCAs. This analysis identified closely related human counterparts for all immunoglobulin gene segments used by the rhesus bNAb UCAs, with average amino acid identities of 90% (V_H_) and 91% (V_K_) (Fig. 3A, D, fig. S10). These included gene segments used by canonical human V3-glycan bNAbs: IGHV4-34 (used by PCDN-38A) and IGHV4-59 (used by PGT121) (Fig. 3A, fig. S10) (14, 17, 34). Additionally, of the 81 V-encoded bNAb contact residues, 50 were biochemically similar (Fig. 3E) and 41 were identical (fig. S11) to the closest human allele. Thus, humans should be able to generate V3-glycan bNAbs akin to those described here, with similar developmental trajectories to breadth and potency.

### Env evolution in the V1 loop precedes bNAb induction

Plasma virus has a lifespan of ~1 hour, and the cells producing it have a lifespan of ~1 day, making the genetic composition of the circulating virus (i.e. quasispecies) a sensitive and dynamic reflection of viral fitness and selection pressures, including those exerted by neutralizing antibodies (41, 50–52). We thus conducted longitudinal single-genome sequencing (SGS) of plasma viral RNA, which provides a direct and proportional representation of target viral sequences and maintains sequence linkage across complete gp160 *env* genes, thereby enabling antibody pressure to be mapped across Env (53, 54).

**Fig. 4.**
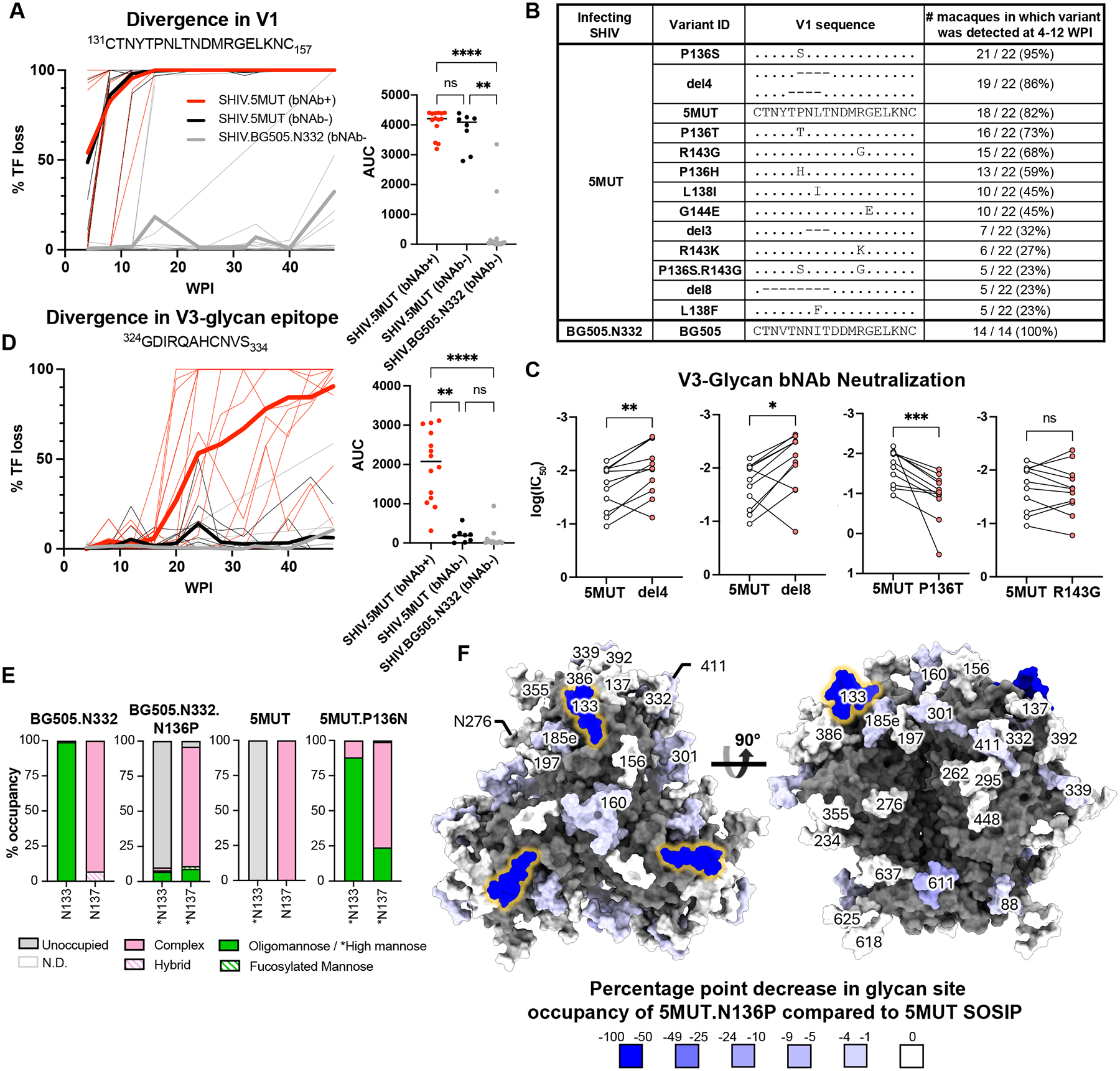
Selection in the V1 region precedes selection in the V3 region in SHIV.5MUT-infected macaques. **(A, D)** Left: Fraction of amino acid variants in the V1 **(A)** or V3 **(D)** regions of Env plotted over time for SHIV.5MUT-infected macaques that did (red) versus did not (black) develop bNAbs, as well as for SHIV.BG505.N332-infected macaques (grey). Thin lines indicate data from individual animals, and thick lines represent group averages. Right: Area under the curve (AUC) for each group. Each dot represents an individual macaque and horizontal lines indicate median. Kruskal-Wallis test with Dunn’s correction for multiple comparisons was used. Not significant (ns) P > 0.05; *P < 0.05; **P < 0.01; ***P < 0.001; ****P < 0.0001. **(B)** V1 sequence variants appearing in at least 20% of macaques between 4–12 weeks post-infection are shown. **(C)** Neutralization potency of representative V3-glycan bNAbs against common V1 variants, shown as log(IC_50_) in μg/mL. Each point represents a single macaque with lines connecting paired samples. Wilcoxon matched-pairs signed-rank test was used. Not significant (ns) P > 0.05; *P < 0.05; **P < 0.01; ***P < 0.001. **(E)** Mass spectrometry-based site-specific glycan analysis of MD39-stabilized 5MUT and BG505.N332 SOSIP trimers. Glycan compositions are grouped into their corresponding categories, with complex-type glycans displayed in pink, oligomannose in green, and unoccupied in gray. For sites in which the site-specific data was obtained with glycosidase digestion by Endo H followed by PNGase F in H_2_O^18^ (indicated by asterisks), the peptide modifications that correspond to distinct glycan types are grouped into high mannose in green, fucosylated mannose in hatched green, complex in pink, and unoccupied in gray. Glycan sites that could not be determined are denoted as “N.D.” **(F)** A glycosylated model of 5MUT SOSIP was generated using the 5MUT-3fill structure, and a heat map of the percentage point change in glycan site occupancy in 5MUT.N136P versus 5MUT was generated and plotted on the Env model. The N185h glycan is not modelled due to a lack of structural determination of the underlying protein sequence covering this region. Man_5_GlcNAc_2_ glycans were modelled at all other PNGS within the structure using GlycoShape Re-Glyco (100) and ChimeraX (90). AUC, area under the curve.

To identify Env variants responsible for priming V3-glycan bNAb precursors, we analyzed the viral quasispecies over time starting shortly after infection. Coincident with the rapid development of high-titer autologous neutralizing antibodies in the plasma (Fig. 1C), we observed striking selection within the V1 loop of Env in all macaques infected with SHIV.5MUT, but in none infected with SHIV.BG505.N332 (Fig. 4A, fig. S12). Mutations were detected as early as week 4 post-infection, leading to complete replacement of the infecting SHIV.5MUT V1 loop sequence by week 16 in all animals (Fig. 4A, fig. S12). Shared patterns of mutations were observed across SHIV.5MUT-infected animals, including a P136S_gp120_ variant present in 21/22 macaques and Δ135–138 / Δ136–139 (del4) deletion variants encoding identical V1 amino acid sequences present in 19/22 macaques (Fig. 4B, fig. S12). Other mutations shared by multiple macaques included Δ137–139 (del3), Δ132–139 (del8), and R143G/K_gp120_ (Fig. 4B, fig. S12).

Hypothesizing that a subset of these V1 variants primed the V3-glycan bNAb lineages, we generated representatives as infectious Envs and assessed their neutralization sensitivity to mature V3-glycan bNAbs. We reasoned that enhanced sensitivity would indicate greater epitope accessibility and thus greater priming potential. Indeed, variants with shortened V1 loops such as del4 and del8 were neutralized with greater potency than the parental SHIV.5MUT. In contrast, the P136T_gp120_ variant was more resistant to neutralization and the R143G_gp120_ variant showed no difference (Fig. 4C). Consistent with the hypothesis that early, shortened-V1 variants like del4 and del8 primed the bNAb lineages, we observed bNAb escape mutations in the V3-glycan epitope and accrual of plasma breadth shortly after their emergence. Specifically, we observed changes in the ^324^GDIR_327_ motif and at H330_gp120_, as well as deletion or shifting of the N332_gp120_ glycan, starting as early as week 16 post-infection in SHIV.5MUT-infected animals that went on to develop plasma breadth (Fig. 4D). In contrast, SHIV.BG505.N332-infected macaques showed virtually no V1 and V3 selection throughout the first 48 weeks of infection (Fig. 4A, B, and D, fig. S12).

The rapid V1 selection in SHIV.5MUT- but not SHIV.BG505.N332-infected macaques suggested that the four distinguishing residues in the 5MUT Env enhance its immunogenicity (Fig. 1B). BG505.N332 contains potential N-linked glycosylation sites (PNGSs) within the V1 loop at positions N133_gp120_ and N137_gp120_ (55, 56), comprising part of the glycan shield that dampens Env immunogenicity (57, 58). While 5MUT retains both of these PNGSs (23), the proline at position 136_gp120_ could alter glycosylation at the adjacent N133_gp120_ and/or N137_gp120_ sites, as proline residues immediately before or after a PNGS can reduce N-glycan occupancy (30, 59, 60). Glycan analysis by mass spectrometry confirmed that the N133_gp120_ PNGS in 5MUT SOSIP was almost completely glycan-devoid, while the N137_gp120_ PNGS remained fully occupied (Fig. 4E–F, fig. S13–14). Similarly, introducing an N136P_gp120_ substitution in BG505.N332 SOSIP substantially reduced glycan occupancy at N133_gp120_ but not at N137_gp120_ (Fig. 4E, fig. S13–14). Reduced V1 glycosylation should enhance 5MUT immunogenicity compared to the parental BG505.N332 Env and induce a targeted humoral response causing V1 selection. Indeed, most of the V1 variants shared among macaques eliminated the P136_gp120_ residue by point mutation or deletion (Fig. 4B, fig. S12), thereby restoring V1 glycosylation at N133_gp120_ and enabling virus escape from the early plasma neutralizing response.

### SHIV.5MUT induces V3-glycan bNAbs via a two-step mechanism

**Fig. 5.**
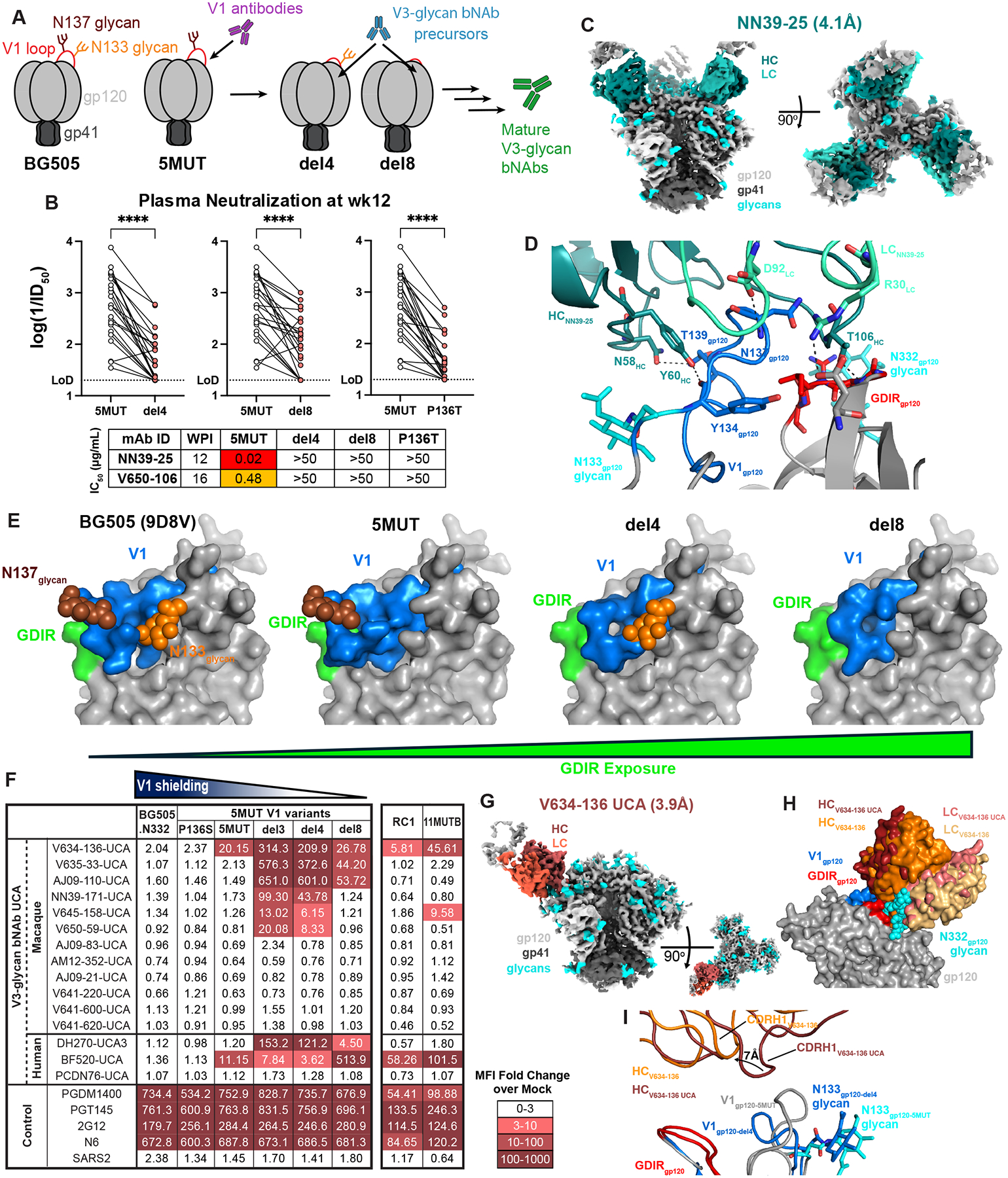
SHIV.5MUT elicits V3-glycan bNAbs via a two-step process. **(A)** Schematic model of SHIV.5MUT-mediated V3-glycan bNAb elicitation. **(B)** Top, neutralization of the indicated V1 variants by plasma from all 24 SHIV.5MUT-infected macaques at week 12 post-infection, with titers represented as log(1/ID_50_). Each point represents a single macaque with lines connecting paired samples. Wilcoxon matched-pairs signed-rank test was used. ****P < 0.0001. Bottom, neutralization activity of two V1-directed monoclonal antibodies, represented as IC_50_ in μg/mL. **(C)** Cryo-EM map of V1-directed antibody NN39-25 in complex with 5MUT-3fill SOSIP. **(D)** Potential interactions between NN39-25 and the ^324^GDIR_327_ motif (red) and V1 loop (blue) of the 5MUT gp120. **(E)** Cryo-EM structures of BG505 (PDB: 9D8V), 5MUT-3fill, del4-3fill, and del8-3fill, highlighting structural differences in GDIR motif exposure as influenced by V1 loop length and glycosylation. **(F)** UCA binding to cell-surface expressed Envs. mRNA encoding each indicated construct was transfected into 293F cells and binding to cell-surface-expressed Env was assessed by flow cytometry. Binding is quantified as fold-change in mean fluorescence intensity (MFI) over mock-transfected cells, and data points with a >3-fold increase are colored as indicated. The results shown are the average of two independent experiments. (**G)** Cryo-EM map of V634-136-UCA in complex with del4-3fill SOSIP. **(H-I)** Structural overlay of V634-136 UCA and mature bNAb revealing nearly identical approach angles **(H)** and a shift in CDRH1 to accommodate the longer V1 loop in 5MUT **(I)**. UCA, unmutated common ancestor; MFI, mean fluorescence intensity; PNGS, potential N-linked glycosylation site.

Given that V1 selection preceded bNAb induction and V3 epitope selection in SHIV.5MUT-infected animals, we hypothesized that bNAb elicitation occurs by a two-step process. Specifically, we propose a model whereby an initial wave of V1-directed antibodies exerts selective pressure on the V1-glycan-deficient 5MUT Env, prompting escape mutants that shorten the V1 loop and expose the underlying V3-glycan epitope, which in turn prime V3-glycan bNAb precursor B cells (Fig. 5A). To test this model, we mapped the earliest plasma response against viruses expressing 5MUT-derived V1 mutant Envs including del4, del8, and P136T_gp120_. In all instances, we observed a reduction in neutralization activity against these variants compared to the parental SHIV.5MUT, confirming that much of the early neutralizing response is V1-directed (Fig. 5B). We also isolated V1-directed mAbs from two macaques that recapitulated this early plasma activity (Fig. 5B). Cryo-EM analysis of a V1-directed Fab (NN39-25) complexed with 5MUT trimer (Fig. 5C) revealed interactions with multiple V1 residues including ^134^YTPNLTN_140_, which contains the four 5MUT substitutions and would therefore be exposed by reduced N-glycan occupancy at position N133_gp120_ (Fig. 5D). Structural overlay with NN39-171, a V3-glycan bNAb isolated from the same animal, revealed that NN39-25 interacted with the V1 loop, whereas NN39-171 exhibited a shifted binding pose favoring interactions with ^324^GDIR_327_ and the N332_gp120_ glycan (fig. S15).

Having shown that induction of V1-directed antibodies like NN39-25 led to the selection of V1 variants *in vivo*, we characterized the variant Envs themselves. We focused on the del4 and del8 variants because viruses bearing these Envs exhibited enhanced neutralization sensitivity to V3-glycan bNAbs (Fig. 4C), suggesting they may provide increased epitope accessibility enabling UCA binding. Additionally, the V1 loop directly overlies the V3-glycan epitope in the pre-fusion Env conformation (61, 62), suggesting that shortening V1 increases epitope exposure. Cryo-EM analysis of unliganded 5MUT, del4, and del8 trimers revealed that smaller footprints of the del4 and del8 V1 loops result in greater exposure of the V3-glycan epitope, including the conserved ^324^GDIR_327_ motif, compared to BG505 and 5MUT (Fig. 5E, Data S6). Reduced V1 glycosylation in 5MUT likely also increases accessibility of the V3-glycan epitope compared to BG505, as evidenced by mass spectrometry (fig. S13–14), although this could not be demonstrated in our cryo-EM structures as the N133_gp120_ and N137_gp120_ glycans were not well-ordered (coordinates could not be assigned past the initial GlcNAc residue) (fig. S16).

### Identification of early Env variants that primed V3-glycan bNAb lineages

We identified del4 and del8 as the most likely V1 Env variants to have engaged diverse V3-glycan bNAb UCAs *in vivo* based on sequencing (Fig. 4B), structural (Fig. 5E), and functional (Fig. 4C) data. We thus generated a stabilized, mRNA-encoded version of each, as well as other V1 variants for comparison (del3, 5MUT.P136S) and controls (5MUT, BG505.N332) (fig. S1A). These Env variants were expressed on the surface of 293F cells and assessed for their ability to bind human (Data S7) and macaque V3-glycan bNAb UCAs (Fig. 5F). All constructs expressed high levels of well-formed Env trimers on the cell surface, as evidenced by robust binding of V2-apex bNAbs that recognize quaternary epitopes (Fig. 5F). Consistent with their full-length, glycosylated V1 loops, neither BG505.N332 nor 5MUT.P136S exhibited detectable binding to any UCA tested, and V1-glycan-deficient 5MUT showed only low-level binding to 2 of 15 UCAs. Similarly, the V1-glycan-deficient immunogens RC1 and 11MUTB bound 2 and 3 UCAs, respectively. In contrast, Env variants that had both short V1 loops and N-glycan deletions (del3, del4, and del8) bound up to 8 of 15 UCAs and did so more robustly than did RC1, 11MUTB, and 5MUT (Fig. 5F). Thus, these shortened-V1 Envs, or others like them, likely primed V3-glycan bNAb precursors *in vivo*.

We determined a cryo-EM structure of V634-136-UCA Fab/del4 trimer complex (Fig. 5G, Data S6), demonstrating that the UCA targeted the V3-glycan epitope with a near-identical angle of approach (Fig. 5H) and nearly all of the same interactions (fig. S17A) as the mature V634-136 bNAb. These results are consistent with V634-136-UCA having acquired only three somatic mutations in its binding interface over the course of affinity maturation (Fig. 3A, fig. S17B). The main structural difference was that the CDRH1 loop of the mature bNAb was shifted by ~7Å away from gp120 compared to that of the UCA, likely serving to accommodate the longer V1 loop in 5MUT compared to del4 (Fig. 5I).

Six macaque UCAs, including AM12-352-UCA and AJ09-83-UCA, did not exhibit detectable binding to any SHIV.5MUT V1 escape variant. To identify Env variants that could have primed these lineages *in vivo*, we produced intermediate mAbs from various stages of lineage maturation by pairing heavy and light chains inferred from the longitudinal BCR sequence dataset (fig. S6, fig. S18A). We detected robust Env binding to early inferred ancestor (iA) mAbs from both lineages: AJ09-83-iA1 bound del3 and AM12-352-iA2 bound 5MUT, and binding magnitude increased with maturity of the lineage members (fig. S18B). Although no binding of del3 and 5MUT to AJ09-83-UCA or AM12-352-UCA was observed, the detection limit of the flow-based assay may be too low to capture physiologically relevant binding that could be enhanced by avidity effects in a germinal center. Given the minimal Env diversity in the circulating virus quasispecies at the time these lineages were initiated (week 4–12) (fig. S12), it is likely that the shortened-V1 Envs in Fig. 4B, or similar ones, were responsible for priming V3-glycan bNAb lineages.

### Env-antibody coevolution guides immunogen design

In humans, HIV-1 Env-bNAb coevolution is a race in which bNAb lineage members exert pressure on the contemporaneous viral quasispecies, leading to the selection of viral escape variants that in turn select for maturing bNAb lineage members capable of binding to them (18, 63). These escape variants often include amino acids that are common in globally circulating HIV-1 strains, and bNAb lineages incrementally acquire heterologous neutralization breadth as they mature to accommodate them (18, 63). To explore whether this conceptual framework applies to our model, we analyzed Env sites under selection in SHIV.5MUT- and SHIV.BG505.N332-infected macaques using LASSIE (64). This analysis identified a total of 78 sites under selection (summary in fig. S19A with analyses of individual animals in fig. S20–33). Of these, 51 sites were found to be under selection in more than one animal, most of which could be attributed to strain-specific escape mutations in known, immunodominant BG505.N332 epitopes including the N241_gp120_/N289_gp120_ glycan hole and the C3-V4-V5 region (fig. S19B).

**Fig. 6.**
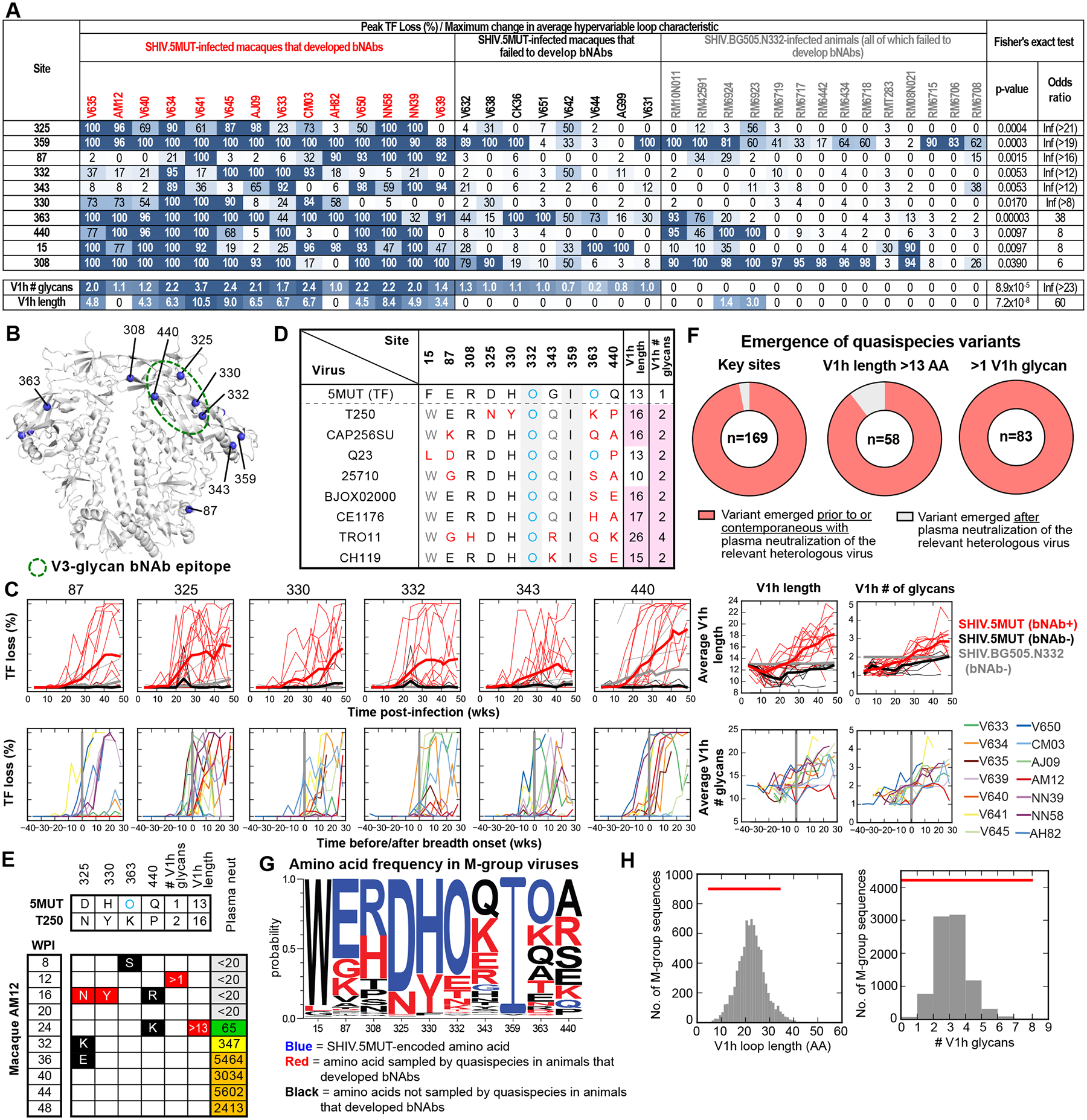
Env-antibody coevolution analysis reveals key Env mutations enriched in SHIV.5MUT macaques that develop bNAbs. **(A)** LASSIE-selected sites that are enriched macaques that developed bNAbs. Numbers indicate peak percentage of transmitted founder (TF) loss. Maximum increase in average number of V1h glycans and V1h length is also shown. **(B)** Selected sites enriched in macaques that developed bNAbs were structurally mapped onto the BG505 Env (PDB ID: 9EHL). **(C)** Top: Mutation frequency (% TF loss) at the indicated sites and average V1h loop length and number of PNGSs. Thin lines indicate data from individual animals and thick lines represent group averages. Bottom: Same data as above, with timepoint of first plasma breadth set to zero weeks (wks). Plots for the remaining four selected sites (15, 308, 359, 363) are shown in fig. S19C. **(D)** Amino acid residues in the heterologous virus test panel that match (black) or do not match (red) SHIV.5MUT. PNGSs are represented as a blue “O”. V1h loops that are longer or have more PNGS than SHIV.5MUT are highlighted in pink. Sites 332 and 359 (highlighted in grey) were invariant and could not be evaluated. **(E)** The subset of the ten bNAb-associated sites that are under selection in macaque AM12 and also differ between T250 and SHIV.5MUT is shown. For each site, both the time point at which mutations are first detected and the sampled residues are indicated. Red shading indicates the sampled residue matches that of the heterologous virus, black shading indicate the sampled residue does not match the heterologous virus, and blue font indicates a PNGS. T250 has a longer V1h loop and more V1h PNGSs than SHIV.5MUT and the timepoint at which the evolving quasispecies first showed increased V1h length or number of PNGSs is shown. Serial plasma neutralization activity against T250 is listed as reciprocal ID_50_. WPI, weeks post-infection. **(F)** Pie charts depict the frequency with which key quasispecies variants (containing mutations at key sites, elongated V1h loops, or additional V1h glycans) emerged prior to or contemporaneous with acquisition of plasma neutralization against the relevant heterologous viruses. Numbers in the center represent the total instances across all 14 SHIV.5MUT-infected macaques that developed bNAbs and all eight heterologous viruses. **(G)** Amino acid frequency in global M-group viruses at the ten key sites enriched in SHIV.5MUT-infected macaques that developed bNAbs. Blue font indicates amino acids encoded by SHIV.5MUT, red font indicates amino acids that were sampled by the quasispecies of macaques that developed bNAbs, and black font indicates amino acids that were not sampled. **(H)** Distribution of global M-group V1h loop lengths (left) and number of glycans (right). Horizontal red lines show the range of V1h length or number of glycans sampled across all SHIV.5MUT-infected macaques that developed bNAbs cumulatively. When two V1 PNGSs were separated by a single proline residue (as in 5MUT), only one was counted as glycosylated.

To identify mutations that could have contributed to V3-glycan bNAb maturation, we focused on ten LASSIE-selected sites enriched in macaques that developed bNAbs (Fig. 6A). These sites included V3-glycan epitope residues 325_gp120_, 330_gp120_, and 332_gp120_, as well as residue 440_gp120_, which is in close proximity to the ^324^GDIR_327_ motif (Fig. 6B) and is a known signature site for human V3-glycan bNAbs (20). We also found that insertions and additions of PNGSs in the hypervariable portion of the V1 region (V1h; residues ^132^TNYTPNLTNDMRG_152_) were more frequent in macaques that developed bNAbs compared to those that did not (Fig. 6A).

Because neutralization breadth is acquired incrementally, Env mutations that are temporally correlated with its onset and enhancement may be important for bNAb maturation. Of the ten selected sites enriched in macaques that developed bNAbs, mutations at gp120 residues 87, 325, 330, 332, 343, and 440 arose either immediately prior to or shortly after the first detection of plasma neutralization breadth. Similarly, increases in V1 length and number of PNGSs were observed around the time of breadth acquisition (Fig. 6C and fig. S20–33). In contrast, mutations at the remaining sites (15_gp120_, 308_gp120_, 359_gp120_, and 363_gp120_) were not temporally associated with breadth development (fig. S19C), although they may be relevant as they were present in the host quasispecies as the bNAb lineages matured (fig. S20–33).

To test whether certain mutations and V1h modifications restrict heterologous neutralization until they are sampled by the viral quasispecies, we determined which of the ten selected sites and V1h features differed between 5MUT and each of the eight viruses in our screening panel (Fig. 6D). Key sites were defined as the subset of positions that differed in the heterologous virus and were under selection in the corresponding animal. We reasoned that amino acid variants at these sites must be recognized by the maturing antibody lineage to gain neutralization activity against the relevant heterologous virus. Sites 332_gp120_ and 359_gp120_ were invariant in our heterologous panel and thus could not be evaluated.

We then compared the timeframe in which mutations emerged at these key sites to the acquisition of neutralization breadth (example in Fig. 6E, summary in fig. S34, analyses of individual animals in fig. S20–33, and detailed description in Supplementary Text). In 164 of 169 cases, mutations at the relevant key sites were observed prior to or concurrent with the acquisition of plasma neutralization against the respective heterologous virus (Fig. 6F, fig. S34). Mutations away from the neutralization-sensitive 5MUT-encoded amino acid in the quasispecies often yielded the exact resistant amino acid encoded by the heterologous virus, further supporting the assumption that accommodation of these specific residues by the maturing bNAb lineages contributes to breadth acquisition (fig. S20–33). Indeed, much of the heterologous M-group diversity at the ten key sites was captured by the quasispecies of SHIV.5MUT-infected animals that developed bNAbs, particularly at residues within the V3-glycan epitope (Fig. 6G). Additionally, the host quasispecies sampled V1h insertions in 52 of 58 instances and V1h PNGS additions in 83 of 83 instances before heterologous plasma neutralization activity was observed (Fig. 6F, fig. S34). Indeed, V1 loop length and number of PNGSs continued to increase over time in macaques that developed bNAbs but not in those that failed to develop bNAbs (Fig. 6C), thereby sampling features that are represented in a greater fraction of globally circulating viruses (Fig. 6H). Overall, emergence of mutations at the key sites was gradual and plasma-level recognition of heterologous viruses was almost never detectable until mutations at all relevant key sites were observed (fig. S34).

## Discussion

A major roadblock to HIV vaccine development efforts has been the lack of a tractable model of bNAb induction in outbred animals. To date, the only example of consistent bNAb elicitation has been fusion peptide-directed bNAbs in rodents (65) and macaques (52), although these were generally of low potency and slow to develop. Here, we address this gap with SHIV.5MUT, which induced V3-glycan bNAbs in 14 of 22 macaques within a year of infection. Plasma neutralization titers reached clinically protective thresholds in many animals, frequently exceeding 1:1000 (ID_50_), and isolated bNAbs achieved up to 68% breadth on a large global virus panel. These results can serve as a benchmark for ongoing vaccine studies and inform the design of next-generation immunogens.

SHIV.5MUT infection induced bNAb lineages by a two-step process: elicitation of an early wave of V1-directed antibodies that selected for Env variants with shortened and hypoglycosylated V1 loops, which in turn engaged and expanded V3-glycan bNAb precursor B cells due to increased epitope accessibility. For sequential immunogen design, this model predicts that the first step of eliciting V1-targeting antibodies can be omitted, and instead V3-glycan bNAb precursors can be primed directly by shortened-V1 Env variants like del4 and del8. Indeed, these variants bound multiple, diverse rhesus and human V3-glycan bNAb precursors, indicating they can serve as lineage-agnostic priming immunogens. Thus, when translating the findings of this study to HIV-1 vaccination regimens, we propose priming with del4 or del8 immunogens, boosting with autologous Env variants incorporating mutations at the ten key sites and two V1h modifications shown to be associated with breadth acquisition, and finally boosting again with a cocktail of naturally-occurring heterologous Envs that include diversity at these same sites. A preclinical trial in macaques testing these concepts is currently underway.

A key finding of the current study was the extraordinary immunogenetic and structural diversity of SHIV.5MUT-elicited bNAbs. Rhesus bNAbs utilized a wide range of immunoglobulin genes, encoded CDRH3 loops ranging in length from 14–25 amino acids, approached Env from a variety of angles, and in some instances did not depend on the N332_gp120_ glycan for recognition and neutralization. Given that human and rhesus antibody repertoires are comparable in size and diversity (66), many of the same or analogous solutions to V3-glycan epitope recognition documented here should be available in humans. The ability to induce such diverse responses would be a desirable property in a vaccine, as it would maximize effectiveness in subjects with heterogeneous immunoglobulin repertoires. Conversely, if SHIV.5MUT infection had led to a single, stereotypical bNAb solution, then elicitation of similar antibodies in humans would require certain immunogenetic features to be widespread in the human repertoire. For example, the CD4bs immunogen eOD-GT8 requires permissive V_H_1-2*02 or *04 alleles, and clinical trial participants lacking these were non-responders (67).

The fact that SHIV.5MUT rapidly induced diverse bNAbs in 64% of macaques – and in two animals primed three distinct bNAb lineages – indicates that authentic V3-glycan bNAb precursors are more abundant than previously appreciated. Other investigators have developed rules to define putative precursors that are based on immunogenetic similarities with a particular UCA, often yielding vanishingly small estimates of precursor frequency in the naïve B cell repertoire (30, 68). In contrast, SHIV.5MUT infection consistently elicited diverse bNAbs with widely varied immunogenetic features, suggesting that current precursor definitions for V3-glycan bNAbs are overly restrictive. Additionally, our data demonstrate that antibodies can converge on similar binding modes despite using distinct germline building blocks, as exemplified by rhesus bNAbs AJ09-83 and V634-136, which bind Env with similar poses but are derived from different V_H_ gene segments. Overall, priming a diverse array of bNAb precursors can serve as a metric of success of current and future vaccine trials.

Our results support the use of V1-modified immunogens to stimulate V3-glycan bNAb precursors in human studies. The CH848.1017.DT Env immunogen, which recently entered human clinical trials (HVTN307 / NCT05903339), was engineered to prime DH270-like bNAb lineages and has a short, glycan-deficient V1 loop (18, 27). This design derived from HIV-infected human subject CH848, who developed potent V3-glycan bNAbs following an initial wave of V1-targeting antibodies that selected for V1-deleted viral variants (18). Two macaques infected with the Env-matched SHIV.CH848 mirrored this pattern of V3-glycan bNAb induction (69). Other immunogens designed to elicit BG18-like antibodies (29, 30) and non-canonical V3-glycan bNAbs (70) have heavily engineered V1 loops that also lack N-glycans, and some of these have advanced to human clinical trials (HVTN144 / NCT06033209) (29, 30). Our findings suggest that the goal of these immunogen platforms should be to prime diverse V3-glycan bNAb precursors. More broadly, sterically increasing epitope exposure by N-glycan elimination and/or shortening of hypervariable loops may serve as a generalizable strategy for inducing bNAbs against HIV-1 and other viruses. Indeed, we recently showed that structure-guided deletion of N-glycans adjacent to the CD4 binding site markedly enhanced elicitation of bNAbs to this epitope in a SHIV model (71).

A limitation of this study is that not all SHIV.5MUT-infected animals developed V3-glycan bNAbs despite shortened-V1 Env variants with priming potential being present in most. This may be due in part to insufficient antigen drive since macaques that did not develop bNAbs had lower plasma viral loads, as found in other studies (69, 72, 73). Immunobiological complexities inherent in using SHIV infection to deliver antigen may also contribute (69). Nevertheless, these limitations also represent opportunities to investigate strategies to improve the consistency, breadth, potency, and durability of V3-glycan bNAb elicitation in a well-defined, outbred primate model.

## Materials and Methods

### Non-human primates

Indian rhesus macaques were housed at Bioqual, Inc., Rockville, MD, in accordance with guidelines of the Association for Assessment and Accreditation of Laboratory Animal Care (AALAC) standards. Care and attention were given to animal husbandry and the physical and psychological well-being of the monkeys, and housing and environmental conditions were maintained as specified by the AALAC. Where possible, animals were co-housed to maintain prior bonding relationships. All procedures involving monkeys followed written standard operating procedures developed in accordance with guidelines of the American Veterinary Medical Association (AVMA), including anesthesia and euthanasia. Throughout the study, attention was given to the minimization of pain and distress, including appropriate administration of analgesics. Clear written clinical endpoint indications for euthanasia were followed, including prespecified weight loss, inappetence, weakness, inability to obtain food or water, infections unresponsive to antibiotics, and signs of severe organ dysfunction. Euthanasia was performed using methods consistent with AVMA guidelines. All experiments were approved by the Institutional Animal Care and Use Committees (IACUC) of the University of Pennsylvania and Bioqual, Inc.

Animals ranged from 3–21 years of age (Data S1) and were healthy and naïve to HIV and SHIV exposure. Animals in Group 1 were immunized with protein-nanoparticle versions of RC1-3fill (wk −16) and 11MUTB-3fill (wk −8). At each time point, a total dose of 100 μg of immunogen and 750 μg of SMNP adjuvant was given subcutaneously, divided evenly between the four limbs. Four macaques received bolus immunizations (V630, V631, V632, and V633) and four received escalating-dose immunizations (V634, V635, V636, and V637). The escalating dose regimen was administered over the course of two weeks, as described in ref (74). Animals in Group 2 were immunized with membrane-anchored mRNA-LNP versions of RC1-3fill (wk −16) and 11MUTB-3fill (wk −8). A total dose of 100 μg of immunogen was given at each time point, with 25 μg administered subcutaneously in each upper limb and 25 μg administered intramuscularly in each lower limb.

At wk 0, all animals were infected with either SHIV.5MUT (Group 1–3) or SHIV.BG505.N332 (Group 4). All but two SHIV.5MUT-infected animals (AH82, AG99) received an intravenous infusion of 25 mg/kg of anti-CD8α (Nonhuman Primate Reagent Resource MT807R1) or anti-CD8β (Nonhuman Primate Reagent Resource CD8b255R1) 2–3 days prior to SHIV inoculation to transiently deplete CD8 T cells, with the goal of facilitating viral replication *in vivo* (Data S1). All SHIV.5MUT infections were performed intravenously. The SHIV.5MUT inoculum was generated by transfecting 293T cells with the respective infectious molecular clone (50 ng of p27 antigen in RPMI1640 with 10% heat-inactivated fetal bovine serum (FBS)). SHIV.BG505.N332-infected animals were part of an earlier dose-titration study (41, 75) to determine the optimal conditions to achieve productive infection and, as such, inoculum dose, inoculum route, and anti-CD8 treatment varied (Data S1).

### Processing and storage of clinical samples

Blood samples were collected from immunized and infected rhesus macaques into sterile vacutainers containing Anticoagulant Citrate Dextrose Solution A (ACD-A). 40 mL of anticoagulated blood was combined into a sterile 50 mL polypropylene conical tube and centrifuged at 1000 × g for 10 min at 20 °C. The plasma phase was collected, clarified by centrifugation, aliquoted into 1 mL cryovials, and stored at −80 °C. The white blood cell (WBC) and red blood cell (RBC) fractions were combined, resuspended in an equal volume of Hanks’ Balanced Salt Solution (HBSS) without Mg^2+^/Ca^2+^ and containing 2 mM EDTA, and distributed evenly into four sterile 50 mL tubes. The volume of each tube was adjusted to 30mL with HBSS-EDTA (2mM), underlaid with 14 mL of 96% Ficoll-Paque (diluted with HBSS-EDTA), and centrifuged at 725 × g for 25 min at 20 °C with slow acceleration and braking. Cells at the phase interface were collected, transferred to a sterile 50 mL tube, diluted to a final volume of 50 mL with HBSS-EDTA (2mM), and centrifuged at 500 × g for 15 min at 20 °C. The cell pellet was resuspended in 30 mL of HBSS with Mg^2+^/Ca^2+^ and 1% FBS, then centrifuged at 200 × g for 15 min at 20 °C. If heavy RBC contamination was observed, lysis was performed using RBC Lysis Buffer (Biolegend 420301). The cell pellet was resuspended in 30 mL of HBSS with Mg^2+^/Ca^2+^ and 1% FBS, and cell count and viability were determined with acridine orange / propidium iodide (AOPI) staining. Cells were centrifuged at 300 × g for 10 min at 20 °C, resuspended at 5–10 × 10^6^ cells/mL in CryoStor CS5 cell cryopreservation media (Sigma C2999), and aliquoted into 1 mL cryovials (Thermo 374503). Cells were stored in a CoolCell container (Corning 432008) at −80 °C overnight and then transferred to vapor-phase liquid nitrogen for long-term storage. Cells collected from lymph nodes and bone marrow were processed in a similar manner. Lymph nodes were excised and immediately placed in RPMI1640 with 10% FBS on wet ice, cleaned of fat and connective tissue via dissection, quartered, and homogenized through a sterile 100 μm strainer. The homogenized cell suspension was transferred to a 50 mL tube, diluted to a final volume of 30 mL with RPMI with 10% FBS, and subjected to Ficoll density gradient purification as described above. Bone marrow was vigorously resuspended in 20 mL of HBSS without Mg^2+^/Ca^2+^, fat and connective tissue were removed, and the resulting single-cell suspension was purified by Ficoll density gradient purification as described above.

### Protein expression and purification for nanoparticle preparations and structure determinations

Soluble SOSIP.664 versions (76) of BG505, 5MUT, del4, and del8 native-like gp140 Env trimers were expressed with MD39 stabilizing mutations (23) and PNGSs introduced at positions 230_gp120_, 241_gp120_, and 344_gp120_ (“3fill” substitutions) to shield an immunodominant off-target glycan hole (25, 57, 58) as described (77). SpyTagged SOSIP Env trimers were prepared by adding a 16-residue SpyTag003 sequence to the C-terminus of each soluble Env trimer. Soluble SOSIP Envs were expressed by transient transfection in Expi293 cells (Life Technologies). Proteins were harvested from culture supernatants and purified using PGT145 immunoaffinity chromatography followed by size-exclusion chromatography (SEC) on Superose 6 (GE Healthcare) in 20 mM sodium phosphate pH 7.5, 150 mM NaCl (PBS) as described (26). Final protein preparations were stored at 4 °C. Fab fragments corresponding to the AJ09-21, AJ09-83, AJ09-110, AM12-340, AM12-347, AM12-351, AM12-352, NN39-25, V634-136, V634-136 UCA, V645-158, and NN39-171 IgGs were generated as described (25). In brief, Expi293 cells were transiently transfected with plasmids encoding an IgG light chain and the Fab region of an IgG heavy chain including a C-terminal 6x-His tag. Fabs secreted into the culture medium were isolated by nickel-nitrilotriacetic acid (Ni^2^^+^-NTA) affinity chromatography (GE Healthcare) followed by purification via SEC using a Superdex 200 16/60 column (GE Healthcare). Purified Fabs were concentrated and stored at 4 °C in a buffer containing 20 mM Tris pH 8.0, 150 mM NaCl (TBS), and 0.02% sodium azide.

### Preparation of SOSIP-mi3 nanoparticles

SpyCatcher003-mi3 particles were prepared by purification from BL21 (DE3)-RIPL *E. coli* (Agilent) transformed with a pET28a SpyCatcher003-mi3 gene (including an N-terminal 6x-His tag) as described (26). Briefly, bacterial cell pellets were lysed with a cell disruptor in the presence of 2.0 mM PMSF (Sigma) and then spun at 21,000 × g for 30 min and filtered with a 0.2 μm filter. Mi3 particles were isolated by ammonium sulfate precipitation followed by SEC using a HiLoad 16/600 Superdex 200 (GE Healthcare) column equilibrated with TBS pH 8.0. SpyCatcher003-mi3 particles were stored at 4 °C and used for conjugation after passing through a 0.2 μm filter and spinning for 30 min at 14,000 × g at 4 °C. For preparation of SOSIP-mi3 nanoparticles, SpyCatcher003-mi3 was incubated with a 2-fold molar excess (SOSIP protomer to mi3 subunit) of purified SpyTagged SOSIP overnight at room temperature in PBS. Conjugated SOSIP-mi3 particles were separated from free SOSIPs by SEC on a Superose 6 10/300 column (GE Healthcare) in PBS. Fractions corresponding to conjugated mi3 particles were identified by SDS-PAGE. Concentrations of SOSIP-mi3 particles were determined using absorbance at 280 nm as measured on a Nanodrop spectrophotometer (Thermo Scientific).

### mRNA design and production

Plasmids encoding membrane-anchored prefusion-stabilized Env trimers matching RC1 and 11MUTB protein immunogens were designed, cloned (Genscript), and used as a template for *in vitro* transcription (fig. S1A). Nucleoside-modified mRNA immunogens were produced using T7 RNA polymerase (Megascript) with co-transcriptional RNA capping (CleanCap 3OMe, Trilink) and incorporation of N1-methylpseudouridine in place of uridine (Trilink) as previously reported (78). A plasmid-encoded polyadenylation sequence (101 nt) was incorporated to improve mRNA stability within the cell. Contaminating double stranded byproducts were removed with cellulose purification and innate immune sensing was assessed as interferon-alpha release following transfection of monocyte-derived dendritic cells. mRNA was evaluated for appropriate size using gel electrophoresis and stored at −20°C.

### mRNA-LNP synthesis

mRNA-LNPs were prepared in four-component lipid nanoparticles as previously described (78). LNP formulations contain ionizable lipid (proprietary to Acuitas) / phosphatidylcholine / cholesterol / PEG-lipid. Lipid and LNP compositions are described in US patent US9,221,127.

### SHIV and pseudovirus production

The construction of SHIV.BG505.N332 was described previously and includes an S375Y_gp120_ mutation that is required for efficient entry and replication in rhesus CD4 T cells (79). SHIV.5MUT was generated by mutagenizing four gp120 residues (V134Y, N136P, I138L, and D140N) in the plasmid encoding this parental strain using the Q5 Site-Directed Mutagenesis Kit (NEB E0554S). Mutants in this and other backbones were generated using the same strategy. Mutagenized plasmids were sequenced to confirm integrity.

Infectious SHIV stocks were produced as previously described (79). Briefly, 5 × 10^6^ HEK 293T cells (ATCC CRL-3216) were plated in a 100 mm tissue culture dish in 10 mL of Dulbecco’s Modified Eagle Medium (DMEM) supplemented with 10% FBS and 100 U/mL penicillin-streptomycin (Gibco 10378-016) and cultured at 37 °C with 5% CO_2_. After 24 hours, 6 μg of SHIV plasmid DNA (for SHIV generation) or 4.5 μg of HIV-1 backbone (SG3ΔEnv) plasmid plus 1.5 μg of Env plasmid (for pseudovirus generation) was diluted in 500 μL of DMEM, combined with 18 μL of FuGENE 6 transfection reagent (Promega E2692), and added dropwise to the cells. Cells were cultured for 48 hours at 37 °C with 5% CO_2_. Virus-containing media was then harvested and centrifuged at 2000 × g for 8 min at 20 °C. Supernatant was divided into 500 μL aliquots and stored at −80 °C. SHIV infection stocks were quantified by measuring p27 antigen using an ELISA (ZeptoMetrix 0801169). Pseudovirus stocks were titrated in TZM-bl cells (80). Briefly, 1.5 × 10^4^ TZM-bl cells were seeded in flat-bottom 96-well tissue culture plates in DMEM with 10% FBS and 100 U/mL penicillin-streptomycin and cultured at 37 °C with 5% CO_2_. Virus stocks were harvested 24 h later, serially five-fold diluted in DMEM with 6% FBS, 100 U/mL penicillin-streptomycin, and 40 μg/mL DEAE-dextran, and then added to the cells with four technical replicates. 48 h later, cells were fixed in PBS containing 0.8% glutaraldehyde and 2.2% formaldehyde for 10 min, washed three times with PBS, and stained with PBS containing 4 μM magnesium chloride, 4 μM, potassium ferricyanide, 4 μM potassium ferrocyanide, and 400 μg/mL X-gal for 3 hours at 37 °C. Cells were washed three times with PBS and imaged on an ELISPOT analyzer (CTL ImmunoSpot 7.0.34.0 Professional Analyzer DC). Infectious virus titers were determined by averaging the number of spots per virus dilution and dividing by the viral input volume.

### Plasma viral RNA quantification

SHIV plasma viral load was quantified using standardized, quality-controlled real-time PCR assays (Applied Biosystems) at the CLIA-certified, NIH/NIAID-sponsored Non-Human Primate Virology Core Laboratory at the Duke Human Vaccine Institute. High-throughput sample processing and PCR setup was facilitated by use of QIAsymphony SP and QIAgility automation platforms (QIAGEN). Viral RNA was isolated from plasma and reverse-transcribed into cDNA using a target-specific primer. cDNA was treated with RNase, added to a custom real-time PCR master mix containing target-specific primers and a fluorescently-labeled hydrolysis probe, and amplified on a QuantStudio3 (Thermo) real-time quantitative PCR machine. Raw data were quality-controlled (including confirmation of positive and negative controls) and mean viral RNA copies/mL was calculated. The lower limit of quantification of this assay was 62 RNA copies/mL.

### Neutralization assay

Neutralization assays were performed using TZM-bl indicator cells as previously described (79, 80). Briefly, 1.5 × 10^4^ TZM-bl cells were plated in flat-bottom 96-well plates in 100 μL of DMEM supplemented with 10% FBS and 100 U/mL penicillin-streptomycin and cultured at 37 °C with 5% CO_2_. 24 h later, plasma samples were heat-inactivated at 56 °C for 1 h and serially five-fold diluted starting at 1:20. To maintain a constant plasma concentration of 5% v/v across all wells, media was supplemented with 10% human serum (Millipore Sigma H5667) at all but the highest dilution. Alternatively, monoclonal antibodies were serially five-fold diluted starting at 50 or 250 μg/mL in media that was not supplemented with human serum. Virus was diluted to achieve a final multiplicity of infection (MOI) of 0.3 when added to the TZM-bl cells. Virus was incubated with plasma or mAb dilutions at 37 °C for 1 h, and this mixture was then added to the adherent TZM-bl cells and cultured at 37 °C with 5% CO_2_. 48 h later, cells were lysed with 0.5% Triton X-100 in PBS and luciferase activity was quantified using a luciferase assay reagent (Promega E1501) and a BioTek Synergy Neo2 plate reader (Agilent). Animals were considered as having developed a bNAb response if plasma collected within the first 48 weeks of infection neutralized at least 3 of 8 heterologous viruses with ID_50_ titers >1:20. All plasma neutralization assays were performed with two technical replicates.

### Generation of soluble Env trimers for flow cytometry

Stabilized HIV Env trimers were expressed and purified as previously described (81). A V3 epitope knockout construct was generated by introducing D325A_gp120_, R327A_gp120_, and S334A_gp120_ mutations into the Q23 backbone. Plasmids encoding Env trimers with C-terminal AviTags were transfected into HEK293F cells (Thermo R79007) cultured in Gibco FreeStyle 293 Expression Medium (Gibco 12338018) using PEI-MAX 4000 transfection reagent (Polysciences 23765). *E. coli* biotin ligase (BirA) was co-transfected to site-specifically biotinylate the biotin-acceptor AviTag peptide on Env. Cells were maintained at 37 °C with 8% CO_2_ and shaking at 125 rpm. Four days post-transfection, cultures were harvested by centrifugation at 2700 × g for 15 min and clarified supernatants containing secreted Env trimers were collected. Env trimers were purified by affinity chromatography using either agarose-bound *Galanthus nivalis* lectin (GNL; Vector Laboratories AL-1243-5) or Toyopearl AF-Tresyl-650M resin (Tosoh Bioscience 0014472) conjugated to the broadly neutralizing antibody PGT145. Antibody conjugation to the resin was performed according to the manufacturer’s protocol. Eluted proteins were further purified by size-exclusion chromatography (SEC) using a Superdex 200 Increase 10/300 GL column (Cytiva 28-9909-44) equilibrated in PBS or TBS. Biolayer interferometry (BLI) was used to assess the efficiency of biotinylation with an Octet RH96 instrument (Sartorius). Biotinylated Env proteins were diluted in PBS supplemented with 0.1% Tween-20 and analyzed using streptavidin biosensors (Sartorius 18-5019) pre-equilibrated in the same buffer. Successful biotinylation was confirmed by a reproducible and saturable loading response within the expected range for mono-biotinylated proteins. Purified biotinylated Envs were conjugated to streptavidin-BV421 (Biolegend 405225), streptavidin-AF647 (Biolegend 405237), or streptavidin-PE (Invitrogen SA10044) by incubating together at a 4:1 molar ratio at 4 °C.

### B cell isolation

5–10 × 10^6^ cryopreserved PBMCs were thawed and resuspended in 10 mL of RPMI supplemented with 10% FBS, 100 U/mL penicillin-streptomycin, and 50 U Benzonase (Millipore Sigma 70664-3). The cell suspension was centrifuged at 300 × g for 5 min at 4 °C, washed once with PBS, and stained with Zombie-NIR viability dye (Biolegend 77184) for 30 min at 4 °C. Cells were washed with PBS supplemented with 5% FBS and stained for 1 hr at 4 °C with a cocktail of antibodies against CD3 (clone SP34-2, BD 557757), CD8α (clone SK1, Invitrogen 47-0087-41), CD14 (clone 61D3, Invitrogen 47-0149-42), CD16 (clone eBioCB16, Invitrogen 47-0168-41), CD20 (clone L27, BD 335793), and IgG (clone G18-145, BD 550931). For isolation of bulk B cells for lineage tracing analysis, antibodies against IgM (clone G20-127, BD 562618) and IgD (polyclonal, Dako F0189) were also used. For isolation of single, Env-specific B cells, cells were washed once with PBS supplemented with 5% FBS and then were stained for 1 h at 4 °C with 1 μg of BV421-, AF647, and/or PE-conjugated soluble Env trimer in PBS supplemented with 5% FBS. V1-directed antibodies were isolated as 5MUT^++^del4^−^ or 5MUT^++^BG505^−^. All other antibodies were isolated using the Env probes listed in Data S4. Cells were then washed three times with PBS supplemented with 5% FBS, filtered through a sterile 100 μm strainer, and sorted on a BD FACSMelody Cell Sorter. Naïve (CD3^−^CD8α^−^CD14^−^CD16^−^CD20^+^IgM^+^IgD^+^) or memory (CD3^−^CD8α^−^CD14^−^CD16^−^CD20^+^IgM^−^IgD^−^IgG^+^) B cells were sorted in bulk into filtered FBS, centrifuged at 300 × g for 5 min, resuspended in 600 μL of cold RNAzol RT (Molecular Research Center RN190), transferred into 2 mL vials (Sarstedt 72.694.396), and stored at −80 °C. Single, Env-specific B cells (CD3^−^CD8α^−^CD14^−^CD16^−^CD20^+^IgG^+^Env^++^) were sorted into individual wells of a 96-well PCR plate containing 20 μL of lysis buffer containing 0.5 μL RNAse OUT (40 U/μL, Invitrogen 10777019), 5 μL 5X First Strand Buffer (Invitrogen 18080044), 1.25 μL DTT (0.1 M, Invitrogen 18080044), 0.0625 μL IGEPAL CA-630 detergent (Sigma I8896), and 13.25 μL nuclease-free water. Cell lysate was centrifuged at 300 × g for 5 min at 4 °C and stored at −20 °C. Bone marrow plasma cells were sorted in a similar manner, with the following changes. After viability staining cells were washed, resuspended in PBS supplemented with 5% FBS and 1:20 anti-FcR (Invitrogen 14-9156-42), and incubated for 15 min at 4 °C. Without washing, cells were then stained with a cocktail of antibodies against CD3, CD8α, CD14, CD16, CD20, CD102 (clone CBR-IC2/2, Biolegend 328506), and CD31 (clone C31.7, Novus NBP2-33136AF647) (82). Plasma cells (CD3^−^CD8α^−^CD14^−^CD16^−^CD20^−^CD102^+^CD31^+^) were sorted into 20 μL of RPMI supplemented with 10% FBS and 100 U/mL penicillin-streptomycin, counted, and immediately carried forward for single-cell analysis using the 10X Genomics platform.

### Single-cell BCR sequencing from PBMCs

Immunoglobulin heavy (IgH) and light (IgK, IgL) chain genes were amplified as previously described (83). Briefly, single B cell RNA was reverse transcribed by adding 2.3 μL random hexamers (200 ng/μL, Thermo SO142), 2 μL dNTP mix (10 mM, Invitrogen 18427088), 1 μL Superscript III reverse transcriptase (200 U/μL, Invitrogen 18080044), and 0.7 μL nuclease-free water per well and incubating in a thermocycler at 42°C for 10 min, 25°C for 10 min, 50°C for 1 hr, and then 94°C for 5 min. IgH, IgK, and IgL genes were amplified by two rounds of nested PCR, each using 3 μL of template, 5 μL 10X buffer (Qiagen 203207), 1 μL dNTP mix (10 mM, Qiagen 201901), 0.5 μL MgCl_2_ (25 mM, Qiagen 203207), 0.5 μL carrier RNA (1 mg/mL, Qiagen 1017647), 0.75 μL forward primer mix (50 μM per primer), 0.5 μL of each reverse primer (25 μM), 0.4 μL HotStart Taq polymerase (5 U/μL, Qiagen 203207), and 39.7 μL nuclease-free water. PCR conditions were as follows: 95°C for 15 min, then 50 cycles of 95°C for 30 sec, 51°C (Round 1) or 58°C (Round 2) for 30 sec, 72°C for 30 sec, with a final extension of 72°C for 7 min. Amplicons were visualized by agarose gel electrophoresis, Sanger sequenced (Genewiz), and analyzed computationally using IMGT V-QUEST (84).

### Single-cell BCR sequencing from bone marrow-derived plasma cells

A CD102^+^CD31^+^ bone marrow cell suspension was loaded onto a Chromium X instrument (10X Genomics) to generate a single-cell bead emulsion, at a loading concentration for a target recovery of 20,000 cells per reaction. Single-cell RNA-seq libraries were then prepared using Chromium Next GEM Single Cell 5’ Kit v3 bead and library construction kit (10X Genomics). BCR libraries were constructed using Chromium Single Cell Human BCR Amplification Kit (10X Genomics) and macaque-specific primers targeting the constant regions of heavy chain IgM, IgG, and IgA gene isotypes and light chain IgK and IgL genes, as described (85). BCR libraries were indexed using Dual Index Kit TT Set A kit (10X Genomics) and sequenced on an Illumina NextSeq 2000 instrument at a minimum read depth of 5000 reads/cell. Sequencing reads were analyzed using Cell Ranger v7.2 (10X Genomics) and heavy chain BCR sequences were recovered from a total of 21,220 cells. AM12-352 lineage members were identified based on CDRH3 length and amino acid sequence homology, heavy chain V, D and J gene segment usage, and light chain V and J gene segment usage.

### Antibody cloning and expression

Immunoglobulin heavy and light chain variable regions of interest were synthesized and cloned (Genscript) into rhesus IgG1, IgK, or IgL expression vectors immediately upstream of the constant region using AgeI/NheI, AgeI/BsiWI, or AgeI/ScaI restriction sites, respectively, as previously described (83). Recombinant mAbs were expressed by co-transfecting paired heavy and light chain plasmids into 293F cells at a 1:2 ratio using ExpiFectamine transfection reagent (Gibco A14525), purified using rProtein A/Protein G Sepharose Gravitrap kit (Cytiva 28985256), concentrated in 30 kDa centrifugal filters (Sigma UFC903024), and buffer exchanged into PBS. Antibody concentration was measured fluorometrically using a Qubit Protein Assay kit (Invitrogen Q33212).

### Bulk BCR sequencing and lineage tracing

Immunoglobulin heavy and light chain sequences were amplified and analyzed from bulk IgM^+^IgD^+^ or IgG^+^ B cells as previously described (41, 69). Briefly, RNA was extracted using RNAzol RT (Molecular Research Center RN190) according to the manufacturer’s instructions. cDNA was synthesized using 5’ rapid amplification of cDNA ends (5’ RACE) with SMARTer template switching and SuperScript II reverse transcriptase (Invitrogen 18064071) and purified using AMPure XP beads (Beckman Coulter A63881). BCR library construction was performed as previously described (41) using KAPA HiFi HotStart ReadyMix PCR Kit (Roche KK2601) and primers annealing in the IgG, IgK, or IgL constant region. Additional PCR steps were used to append barcodes and Illumina P5 and P7 sequencing adapters. All libraries were sequenced on an Illumina MiSeq sequencer (with 2 × 300 bp runs using the 600-cycle MiSeq Reagent V3 kit) or NextSeq 2000 sequencer (with P1 or P2 600-cycle kits). Sequences from the naïve IgM^+^IgD^+^ B cell population were used to construct an individualized germline immunoglobulin repertoire for each macaque of interest using IgDiscover (46) and MINING-D (86) computational pipelines, as previously described (41). Sequences from the IgG^+^ B cell population were used to trace lineages of interest and infer unmutated common ancestors using the SONAR computational pipeline (47), as previously described (41).

### Identification of homologous human V gene segment alleles

Homologous human V gene segment alleles were found using global alignments between the rhesus UCA V gene segment alleles and the orgdb database (49) after removal of novel alleles (designated with i) with BLOSUM62 scoring and gap opening and extension penalties of 11 and 1, respectively (87). The best human V gene segment allele was identified based on protein identity, and ties were broken using biochemical similarity.

### Viral RNA sequencing

The SHIV *rev-vpu-env* cassette was sequenced using a single-genome sequencing (SGS) approach as previously described (53, 54). Briefly, reverse transcription of RNA was performed using reverse transcriptase (SuperScript III) and reverse primer SIVmac251.R.R1 5′-CACTAGCTTACTTCTAAAATGGCAGC-3′ (nt 10,138–10,163, SIVmac239) according to the manufacturer’s instructions. The *rev-vpu-env* cassette was amplified by nested PCR with the following primers: first round forward primer SHIV.RevEnv.F1 5′-CGAAAGGCTTAGGCATCTCCTATG-3′ (nt 5949–5972, HXB2), second round forward primer SHIV.RevEnv.F2 5′-TTAGGCATCTCCTATGGCAGGAAGA-3′ (nt 5,957–5,981, HXB2), first round reverse primer SIVmac251.R.R1 5′-CACTAGCTTACTTCTAAAATGGCAGC-3′ (nt 10,138–10,163, SIVmac239), and second reverse primer SIVmac251.R.R2 5′- TACTTCTAAAATGGCAGCTTTATTGAAGAGG-3′ (nt 10,125–10,155, SIVmac239). A total of 8,406 *env* gp140 sequences were aligned using the MUSCLE alignment method and manually inspected using Geneious Prime to improve the alignment results based on codon translation. The sequence alignment was then analyzed using Pixel plot (https://www.hiv.lanl.gov/content/sequence/pixel/pixel.html) and the LASSIE program (64).

### Cryo-EM sample preparation

Purified Fabs and SOSIP Envs were incubated at a Fab:Env protomer molar ratio of ~4:1 at room temperature (for mature bNAb Fabs) or a ratio of ~1.1:1 at 37 °C (for V634-136-UCA Fab). Resulting mature Fab/Env complexes were purified by SEC using a Superdex 200 Increase 10/300 GL analytical column (GE Healthcare Life Sciences) in TBS pH 8.0, and fractions corresponding to a Fab/Env complex were concentrated to a final concentration of 2–3 mg/mL using a 50 kDa molecular weight cutoff spin concentrator (Millipore). The V634-136-UCA Fab/Env complex was not subjected to SEC purification.

Immediately prior to grid preparation, a fluorinated octyl maltoside solution (Anatrace) was added to each sample from a 0.5% (w/v) stock to a final concentration of 0.02% (v/v) and 3 μL of each complex was applied to freshly glow-discharged Quantifoil R1.2/1.3 grids (300 mesh Cu; Electron Microscopy Sciences) that had been treated for 1 min at 20 mA using a PELCO easiGlow device (Ted Pella). Grids were plunge-frozen using a Vitrobot Mark IV (Thermo Fisher) at 22 °C and 100% humidity. Blotting was performed with Whatman No. 1 filter paper for 3 s using a blot force of 0. Samples were vitrified by rapid plunging into liquid ethane cooled by liquid nitrogen.

### Cryo-EM data collection

Single-particle cryo-EM data acquisition was performed on two transmission cryo-electron microscopes: a Titan Krios (Thermo Fisher) operating at 300 kV was used for imaging the AJ09-21, AJ09-83, AJ09-110, AM12-351, and V634-136-UCA Fabs in complex with a SOSIP Env, and a Talos Arctica (Thermo Fisher) operating at 200 kV was used for the AM12-340, AM12-347, AM12-352, NN39-25, V634-136, V645-158, and NN39-171 Fabs in complex with a SOSIP Env. The unbound structure of 5MUT-3fill SOSIP was imaged on the Titan Krios, while the unbound structures of del4-3fill and del8-3fill SOSIPs were imaged on the Talos Arctica. Automated data collection was carried out using SerialEM software (88), employing beam-image shift across a 3×3 grid of 1.2 μm holes with one exposure per hole. For the datasets recorded on the Krios, 40-frame movies were captured in super-resolution mode using a Gatan K3 camera positioned behind a BioQuantum energy filter (Gatan) with a 10 eV slit and a super-resolution pixel size of 0.416 Å (105,000x magnification). For datasets collected on the Talos Arctica, 40-frame movies were captured in super-resolution mode using a Gatan K3 camera with a pixel size of 0.435 Å (45,000x magnification). Data processing was carried out for all datasets using cryoSPARC v4 (89). Data collection parameters are summarized in Data S6.

### Cryo-EM data processing

Briefly, cryo-EM movies were patch motion corrected in cryoSPARC v4 (89) to account for beam-induced motion, including dose weighting, following the binning of super-resolution frames. For CTF parameter estimations, non-dose-weighted micrographs were processed using the Patch CTF job in cryoSPARC. Micrographs displaying poor CTF fits or evidence of crystalline ice in their power spectra were excluded from further analysis. Particle picking was performed in cryoSPARC v4 (89) using Blob picker (minimum particle diameter = 120 Å, maximum particle diameter = 180 Å) for reference-free selection. Particle extraction was carried out using a box size of 360 Å. Particles were subjected to several rounds of 2D classification. The best class averages representing different views were used to generate ab initio models, which were further refined using homogenous refinement (using minimize over per-particle scale) or non-uniform refinement by applying C1 or C3 symmetry. The particles and 3D volumes from homogenous refinement jobs were combined with micrographs from the CTF estimation for reference-based motion correction before using homogenous or non-uniform refinement to generate the final refined 3D volume for each structure.

### Cryo-EM structure modelling and refinement

To generate initial coordinates of the complex, individual chains from reference structures of the fully glycosylated BG505 SOSIP.664 HIV-1 Env trimer (PDB: 5T3X or 5T3Z) were independently docked into cryo-EM density maps using UCSF ChimeraX (90). Preliminary models were subjected to rigid-body fitting and subsequently refined in real space against the EM maps. Manual adjustments and sequence corrections were performed using Coot v.0.8.9 (91), followed by iterative cycles of model rebuilding in Coot and refinement using Phenix (92). N-linked glycans were incorporated at predicted glycosylation sites by interpreting low-resolution (“blurred”) maps filtered with varying B-factors. Final models were assessed for stereochemical quality using MolProbity (93).

### Structural analyses

Structure figures were created using ChimeraX (90) and PyMOL (94). To highlight Fab densities within EM reconstructions, maps were segmented, with each region visualized in a distinct color. The gp41 region was rendered using a color scheme that corresponded to the fitted SOSIP coordinates from lower-resolution reconstructions. Estimations of local resolution were performed using cryoSPARC v4 (89). To quantify interfacial interactions, buried surface areas were analyzed via PDBePISA v1.48.65 (95), employing a 1.4 Å solvent probe radius. Hydrogen bonds were defined as interactions involving distances below 4.0 Å and an angle between donor, hydrogen, and acceptor atoms greater than 90°. Van der Waals contacts were considered when interatomic distances were under 4.0 Å. These interaction assessments are considered approximate due to limitations in resolution. Structural comparisons, including root-mean-square deviation (r.m.s.d.) values from pairwise Cα alignments, were calculated in PyMOL (94) without applying outlier exclusion. Criteria used to define epitope regions are provided in the respective figure legends.

The angles of approach of V3-directed antibodies were calculated as described (30). The Env trimer 3-fold symmetry axis was aligned on the z-axis, with the center of mass of one monomer’s V3-glycan epitope placed on the x-axis. The V3-glycan epitope was defined by residues 324_gp120_, 325_gp120_, 326_gp120_, 327_gp120_, 328_gp120_, 329_gp120_, 330_gp120_, 415_gp120_, 416_gp120_, and 417_gp120_. The center of mass of a gp41 residue (587_gp41_ in all three protomers) was aligned to the negative z-axis. The latitudinal angle was that formed by the z-axis and a vector from the V3-glycan epitope center of mass to the HC center of mass (carbon-a atoms for six β-strands; HC residues 21–24, 34–39, 46–52, 67–71, 77–82, and 89–92) in the x-z plane. The longitudinal angle was that formed by the x-axis and the V3-glycan epitope-HC vector in the x-y plane. The HC-LC twist angle was the angle between the x-axis and a vector from the LC center of mass (carbon-α atoms for six β-strands, LC residues 18–24, 34–37, 45–48, 62–66, 70–76, and 84–88) to the HC center of mass in the x-y plane.

### Site-specific glycan analysis

100 μg aliquots of each sample were denatured for 1 h in 50 mM Tris/HCl, pH 8.0 containing 6 M of urea and 5 mM dithiothreitol (DTT). Next, Env samples were reduced and alkylated by adding 20 mM iodoacetamide (IAA) and incubated for 1 h in the dark, followed by a 1 h incubation with 20 mM DTT to eliminate residual IAA. The alkylated Env samples were buffer exchanged into 50 mM Tris/HCl, pH 8.0 using Vivaspin columns (10 kDa) and two of the aliquots were digested separately overnight using Trypsin (Mass Spectrometry Grade, Promega), chymotrypsin (Promega), or alpha-lytic protease (Sigma Aldrich) at a ratio of 1:30 (w/w). The next day, the peptides were dried and extracted using an Oasis HLB μElution Plate (Waters).

The peptides were dried again, resuspended in 0.1% formic acid, and analyzed by nanoLC-ESI MS with a Vanquish Neo (Thermo Fisher Scientific) system coupled to an Orbitrap Eclipse Tribrid mass spectrometer (Thermo Fisher Scientific) using stepped higher energy collision-induced dissociation (HCD) fragmentation. Peptides were separated using a μPAC Neo HPLC Column (180 μm × 110 cm). A trapping column (PepMap 100 C18 3 μM 75 μM × 2cm) was used in line with the LC prior to separation with the analytical column. The LC conditions were as follows: 280-minute linear gradient consisting of 4–32% acetonitrile in 0.1% formic acid over 260 min followed by 20 min of alternating 76% acetonitrile in 0.1% formic acid and 4% ACN in 0.1% formic acid, used to ensure all sample had eluted from the column. The flow rate was set to 300 nL/min. The spray voltage was set to 2.5 kV and the temperature of the heated capillary was set to 55 °C. The ion transfer tube temperature was set to 275 °C. The scan range was 350–2000 m/z. The stepped HCD collision energies were set to 15, 25, and 45% and the MS2 for each energy was combined. Precursor and fragment detection was performed using an Orbitrap at a resolution MS^1^ = 120,000, MS^2^ = 30,000. A standard AGC target for MS^1^ (4e^5^) and MS^2^ (1e^4^) and auto-injection times (MS^1^ =50 ms MS^2^ =54 ms) were used.

Glycopeptide fragmentation data were extracted from the raw file using Byos (Version 5.5; Protein Metrics Inc.). The glycopeptide fragmentation data were evaluated manually for each glycopeptide; the peptide was scored as true-positive when the correct b and y fragment ions were observed along with oxonium ions corresponding to the glycan identified. The MS data was searched using the Protein Metrics 305 N-glycan library. The relative amount of each glycan at each site as well as the unoccupied proportion was determined by comparing the extracted ion chromatographic areas for different glycotypes with an identical peptide sequence. All charge states for a single glycopeptide were summed. The precursor mass tolerance was set at 4 ppm and 10 ppm for fragments. A 1% false discovery rate (FDR) was applied. Glycans were categorized according to the composition detected.

HexNAc(2)Hex(9-3) was classified as M9 to M3. Any of these structures containing a fucose were categorized as FM (fucosylated mannose). Complex-type glycans were classified according to the number of HexNAc subunits and the presence or absence of fucose. Core glycans refer to truncated structures smaller than M3. As this fragmentation method does not provide linkage information, compositional isomers are grouped.

To obtain data for sites that frequently present low intensity glycopeptides, the glycans present on the glycopeptides were homogenized to boost the intensity of these peptides. This analysis loses fine processing information but enables the ratio of high mannose : complex : unoccupied to be determined. The remaining glycopeptides were first digested with Endo H (New England Biolabs) to deplete oligomannose- and hybrid-type glycans and leave a single GlcNAc or GlcNAcFuc residue at the corresponding site. The reaction mixture was then dried completely and resuspended in a mixture containing 50 mM ammonium bicarbonate and PNGase F (New England Biolabs) using only H_2_O^18^ (Sigma-Aldrich) throughout. This second reaction cleaves the remaining complex-type glycans but leaves the GlcNAc residues remaining after Endo H cleavage intact. The use of H_2_O^18^ in this reaction enables complex glycan sites to be differentiated from unoccupied glycan sites as the hydrolysis of the glycosidic bond by PNGaseF leaves a heavy oxygen isotope on the resulting aspartic acid residue. The resultant peptides were purified as outlined above and subjected to reverse-phase (RP) nanoLC-MS. Instead of the extensive N-glycan library used above, three modifications were searched for: +203 Da corresponding to a single GlcNAc, or +349 corresponding to a GlcNAcFuc, a remnant of an oligomannose/hybrid glycan (+/− fucose), and +3 Da corresponding to the O^18^ deamidation product of a complex glycan. Data acquisition and analysis was performed as above and the relative amounts of each glycoform were determined, including unoccupied peptides.

### Longitudinal Env evolution analyses

Our previously established algorithm LASSIE was used to identify Env sites under natural selection in each RM using the threshold of ≥80% TF loss at any timepoint (64), as was done previously for SHIV-infected macaques (69). To identify selected Env sites enriched in macaques that developed bNAbs, the contingency tables of number of macaques that developed or failed to develop bNAbs with or without a site under selection were subjected to Fisher’s exact tests and all associations with uncorrected p < 0.05 were identified. All enriched sites also met Benjamini-Hochberg multiple test correction with false discovery rate (FDR) < 20%. Hypervariable V1 (V1h) loop characteristics of length and number of N-glycans were also tested for statistical enrichment using maximum increase in each characteristic’s timepoint average over that of TF for each macaque. For calculation of the number of V1h PNGSs for a given sequence, if two sequons were separated by a Pro (e.g., NNTPNAT), only one was assumed to be N-glycosylated based on 5MUT site-specific N-glycan analyses. Mutations at LASSIE-selected sites and V3 loop sites were visualized using the Python software Logomaker (96).

### Env-bNAb coevolution analysis

To assess whether plasma recognition of heterologous viruses required quasispecies mutation away from the infecting SHIV.5MUT (termed transmitted founder, or TF, virus) at key sites, we determined how often mutations emerged in the viral quasispecies at these sites prior to or contemporaneously with the earliest observed neutralization of each heterologous virus. Of the ten key sites identified as being preferentially selected in macaques that developed bNAbs (gp120 sites 15, 87, 308, 325, 330, 332, 343, 359, 363, and 440), we determined the subset relevant to recognition of a particular heterologous virus on a per-animal basis. Two of these ten sites (the PNGS at position 332_gp120_ and the I359_gp120_) did not vary in our heterologous panel and, as such, could not be evaluated. We assumed that the bNAb lineage in a given animal would select the most relevant resistance sites, and thus the subsets of sites differ between animals. For each heterologous virus in our screening panel, we only analyzed sites that differed between that virus and SHIV.5MUT, assuming that variation at those sites must be acquired before the bNAb lineage can recognize that virus. Additionally, sites 15_gp120_ and 343_gp120_ were not considered to be relevant to recognition of viruses encoding M-group consensus residues W15_gp120_ or Q343_gp120_, as these residues were never sampled by the quasispecies in any SHIV.5MUT-infected macaque that developed bNAbs and thus are unlikely to be associated with resistance.

For each macaque, we evaluated key sites within the V3 loop (gp120 residues 308, 325, and 330) that were under selection regardless of whether they met the 80% LASSIE cutoff. Key sites outside the V3 loop (gp120 residues 15, 87, 343, 363, and 440) were only evaluated for a given macaque if they met the 80% LASSIE cutoff. We preferred to be inclusive for the key sites within the V3 loop (gp120 residues 308, 325, and 330) given their strong potential to contribute to V3 neutralization sensitivity of viruses, and we wanted to avoid missing their impact on bNAb evolution due to our conservative LASSIE cutoff. We determined the earliest timepoint at which mutations were observed at each of the relevant key sites in the evolving quasispecies of a given animal, even if the mutant was rare at that timepoint. While the mutant residue often matched that carried by the heterologous virus, we interpreted any variant residue as potentially contributing to recognition. A detailed description of this analysis as applied to macaque V634 is described in the Supplementary Text. A summary of all results is shown in fig. S34 and individual analyses for each macaque are shown in fig. S20–33.

### Statistical analyses

Statistical tests were calculated in GraphPad Prism 10 (version 10.4.2) or using the Stats module from SciPy (version 0.18.0) (99).

## Supplementary Material

Supp Info

Data S4

Data S5

Data S1

Data S3

Data S6

Data S2

Data S7

Print summary

Reproducibility checklist

Supplementary Text

Figs. S1 to S33

Data S1 to S7

## Data Availability

All monoclonal antibody isolate sequences are deposited at GenBank under accession numbers PX059660–PX060135 and PX390224–PX391126. BCR repertoire next-generation sequencing data are available under the NCBI BioProject PRJNA1305399. Longitudinal Env SGS gp140 sequences are deposited at GenBank under accession numbers PX297533–PX306283 and MN467402–MN472740. Models and cryo-EM density maps were deposited in the PDB (9YHO, 9YHQ, 9YHR, 9YHS, 9YHT, 9YIB, 9YID, 9YIE, 9YIF, 9YIG, 9YIE, 9YII, 9YIJ, 9YIK, 9YIL) and EMDB (EMD-72969 through EMD-72973 and EMD-72985 through EMD-72994). Additional structural information can be found in Data S6. Mass spectrometry data has been deposited to the MassIVE server (MSV000101102). All other data needed to evaluate the conclusions in the paper are available in the main text or supplementary materials. No code was created in this study. Reagents described in this manuscript including SHIVs, mRNA immunogens, and monoclonal antibodies are available from George M. Shaw under a Material Transfer Agreement with the University of Pennsylvania. Protein-nanoparticle immunogens are available from Pamela J. Bjorkman under a Material Transfer Agreement with California Institute of Technology. SMNP adjuvant is available from Darrell J. Irvine under a Material Transfer Agreement with Massachusetts Institute of Technology.
