# Supplementary material for: Induction of Broadly Neutralizing HIV Antibodies by a Two-Step Mechanism Informs Vaccine Design
Source: Science. Author manuscript; Available in PMC 2026 Jun 26. (PMC13308464; doi:10.1126/science.aec6396)
Supplement: Print summary [file NIHMS2174568-supplement-Print_summary.pdf]

Induction of Broadly Neutralizing HIV Antibodies by a Two-Step Mechanism Informs Vaccine Design

Ashwin N. Skelly†, Harry B. Gristick†, Hui Li†, Edem Gavor†, Andrew J. Connell, Edward F. Kreider, Lorie Marchitto, Michael P. Hogarty, Maddy L. Newby, Joel D. Allen, Weimin Liu, Anthony P. West Jr., Kasirajan Ayyanathan, Mary S. Campion, Kaitlyn Winters, Colette G. Gordon, Rebecca A. Osbaldeston, Macy J. Akeley, Emily Lewis, Yingying Li, Ajay Singh, Kendra Cruickshank, Younghoon Park, Chengyan Zhao, Xuduo Li, Khaled Amereh, Elizabeth Van Itallie, John W. Carey, Amie Albertus, Andrew T. DeLaitsch, Jennifer R. Keeffe, Melinda G. Lituchy, Agnes A. Walsh, Daniel J. Morris, Rumi Habib, Frederic Bibollet-Ruche, Nitesh Mishra, Gabriel Avillion, Nicholas S. Koranda, Samantha J. Plante, Christian L. Martella, Jinery Lora, Eric J. D. Wang, Mark G. Lewis, Malcolm A. Martin, Michel C. Nussenzweig, Michael S. Seaman, Darrell J. Irvine, Kevin J. Wiehe, Barton F. Haynes, Kshitij Wagh, Bette Korber, Raiees Andrabi, Max Crispin, Drew Weissman, Pamela J. Bjorkman*, Beatrice H. Hahn*, George M. Shaw*

Introduction: Broadly neutralizing antibodies (bNAbs) against HIV are thought to be a critical component of both prevention and cure strategies to achieve sustained epidemic control. bNAbs target highly conserved epitopes on the HIV envelope (Env) glycoprotein such as the V3-glycan patch and prevent viral entry into host cells, thereby providing protective immunity. A major roadblock to vaccine and cure research has been the lack of an outbred animal model in which potent bNAbs can be rapidly and reliably induced at clinically protective titers. Such a model would enable dissection of the molecular pathways underlying bNAb elicitation and maturation, ultimately informing iterative vaccine design efforts.

Rationale: Potent bNAbs develop naturally in only a small subset of chronically infected patients, typically requiring months or years for an evolved Env variant to engage a bNAb precursor B cell, followed by complex pathways of affinity maturation to breadth and potency. While studies of Env-bNAb coevolution in humans have informed immunogen design, they are hampered by a paucity of longitudinal sequencing data and may be difficult to generalize given that each infecting viral strain is distinct. The simian-human immunodeficiency virus (SHIV) model overcomes this limitation by enabling detailed analysis of multiple macaques infected by the same virus, although bNAb development is still infrequent and slow in animals infected with SHIVs bearing primary Envs. We hypothesized that we could enhance the consistency and rapidity of bNAb elicitation by designing a SHIV (SHIV.5MUT) presenting an epitope-focused Env that selectively exposes the V3-glycan epitope.

Results: SHIV.5MUT infection rapidly elicited potent V3-glycan bNAbs in 14 of 22 macaques within the first year of infection. In contrast, 0 of 14 animals infected with the parental SHIV.BG505.N332 strain, which differs by only four amino acids in the V1 region of Env, developed bNAbs in the same timeframe. We isolated 12 distinct bNAb lineages from SHIV.5MUT-infected animals that achieved up to 68% neutralization breadth against a 130-virus test panel, on-par with the best human-derived V3-glycan bNAbs. These bNAbs exhibited structural and genetic diversity, utilizing a wide range of approach angles and immunoglobulin genes and exhibiting CDRH3 loop lengths from 14-25 amino acids. Longitudinal B cell receptor sequencing enabled unambiguous inference of 12 bNAb precursors. In parallel, longitudinal viral

Env sequencing revealed a two-step mechanism of bNAb induction that was shared across multiple animals. Specifically, the initial humoral response targeted an exposed V1 region specific to the 5MUT Env and selected for viral escape variants with shortened, hypoglycosylated V1 loops. These mutations increased accessibility of the underlying V3-glycan epitope, thereby enabling the priming of multiple V3-glycan bNAb precursor B cells. Common routes of subsequent Env-bNAb coevolution revealed ten key Env mutations and two V1 loop features that likely drove affinity maturation and acquisition of neutralization breadth.

Conclusion: We describe a model of rapid, consistent V3-glycan bNAb elicitation in outbred macaques. The immunogenetic and structural diversity of these bNAbs, as well as their breadth and potency, can serve as a benchmark for future vaccine trials. The common routes of Env-bNAb coevolution leading to neutralization breadth can be used as a blueprint for next-generation immunogen design.

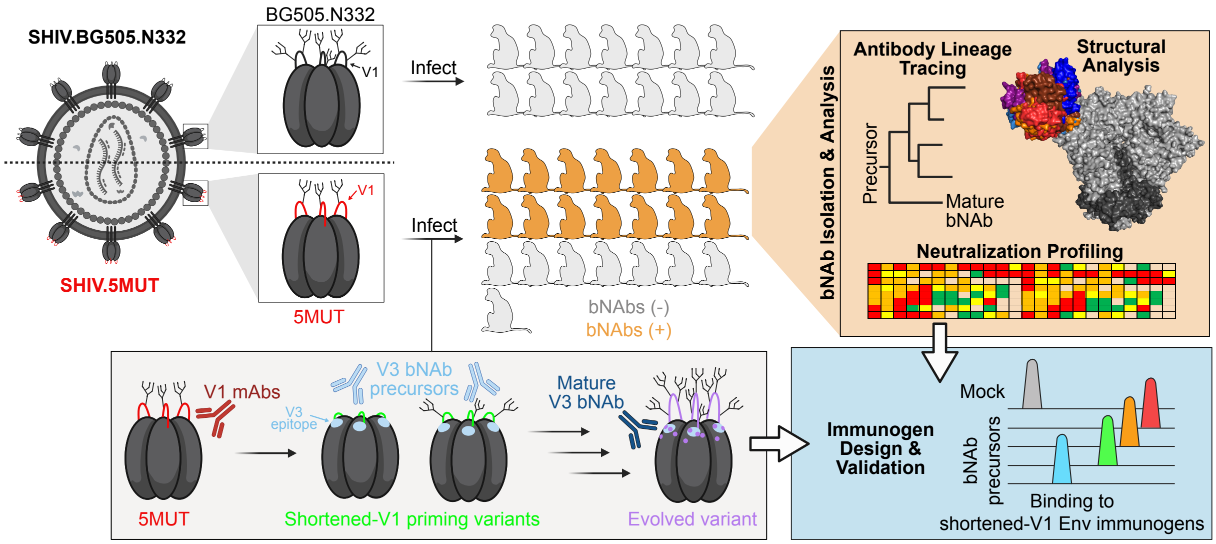

SHIV.5MUT elicits V3-glycan bNAbs in macaques by a two-step mechanism. The 5MUT Env V1 loop is hypoglycosylated compared to BG505.N332. An early wave of antibodies targeting the SHIV.5MUT V1 loop selects for V1-shortened escape variants, which expose the underlying V3-glycan epitope and prime bNAb precursors. V3-glycan bNAbs are immunogenetically and structurally diverse. Env-bNAb coevolution analysis enabled design of priming immunogens that bind multiple bNAb precursors.
