# Supplementary material for: Induction of Broadly Neutralizing HIV Antibodies by a Two-Step Mechanism Informs Vaccine Design
Source: Science. Author manuscript; Available in PMC 2026 Jun 26. (PMC13308464; doi:10.1126/science.aec6396)
Supplement: Supp Info [file NIHMS2174568-supplement-Supp_Info.docx]

Supplementary Materials for

**Induction of Broadly Neutralizing HIV Antibodies by a Two-Step Mechanism Informs Vaccine Design**

Ashwin N. Skelly, Harry B. Gristick, Hui Li, Edem Gavor, Andrew J. Connell, Edward F. Kreider, Lorie Marchitto, Michael P. Hogarty, Maddy L. Newby, Joel D. Allen, Weimin Liu, Anthony P. West Jr., Kasirajan Ayyanathan, Mary S. Campion, Kaitlyn Winters, Colette G. Gordon, Rebecca A. Osbaldeston, Macy J. Akeley, Emily Lewis, Yingying Li, Ajay Singh, Kendra Cruickshank, Younghoon Park, Chengyan Zhao, Xuduo Li, Khaled Amereh, Elizabeth Van Itallie, John W. Carey, Amie Albertus, Andrew T. DeLaitsch, Jennifer R. Keeffe, Melinda G. Lituchy, Agnes A. Walsh, Daniel J. Morris, Rumi Habib, Frederic Bibollet-Ruche, Nitesh Mishra, Gabriel Avillion, Nicholas S. Koranda, Samantha J. Plante, Christian L. Martella, Jinery Lora, Eric J. D. Wang, Mark G. Lewis, Malcolm A. Martin, Michel C. Nussenzweig, Michael S. Seaman, Darrell J. Irvine, Kevin J. Wiehe, Barton F. Haynes, Kshitij Wagh, Bette Korber, Raiees Andrabi, Max Crispin, Drew Weissman, Pamela J. Bjorkman, Beatrice H. Hahn, George M. Shaw

Corresponding author: [bjorkman@caltech.edu](mailto:bjorkman@caltech.edu) (P.J.B.), [bhahn@pennmedicine.upenn.edu](mailto:bhahn@pennmedicine.upenn.edu) (B.H.H.), [shawg@pennmedicine.upenn.edu](mailto:shawg@pennmedicine.upenn.edu) (G.M.S.)

**The PDF file includes:**

Supplementary Text

Figs. S1 to S33

Legends for Data S1 to S7

Supplementary Text

Immunogen design

To retain immunogen Envs in their prefusion conformation, we stabilized protein and mRNA constructs using MD39 mutations (23) and introduced potential N-linked glycosylation sites (PNGSs) at gp120 positions 230, 241, and 344 (“3fill”) to shield immunodominant off-target epitopes (26, 57, 58). Envs expressed by mRNA constructs were further engineered to include an HLA-DR signal peptide, a flexible (GGGGS)_2_ linker in place of the furin cleavage site, two helix-breaking prolines, and a truncated SIVmac cytoplasmic tail with an endocytosis knockout mutation (78) **(fig. S1A)**. Both RC1-3fill and 11MUTB-3fill mRNA constructs expressed well on the surface of 293F cells and exhibited favorable antigenic profiles **(fig. S1B)**. The protein immunogens were multimerized on mi3 nanoparticles and delivered with saponin/MPLA nanoparticle (SMNP) adjuvant as either a bolus (n=4) or an escalating dose regimen over the course of two weeks (n=4) (74). The mRNA immunogens were encapsulated in lipid nanoparticles (LNPs) and delivered as a bolus.

Env-bNAb coevolution

An example of Env-bNAb coevolution analysis as applied to macaque V634 is described here. Nine of the ten key sites are under selection in animal V634 **(Fig. S21)**. Only six of these differ between SHIV.5MUT and T250 **(Fig. 6D)**, of which W15_gp120_ and Q343_gp120_ were deemed to be irrelevant to breadth acquisition as described in **Materials and Methods**. As such, only the four remaining sites (gp120 residues 325, 330, 363, and 440) were considered to be relevant for the bNAb lineage in this animal to recognize T250 **(Fig. S21)**. Consistent with our hypothesis, mutations emerged at all four of these sites before or at the timepoint at which plasma neutralization of T250 was first detected. Indeed, in animal V634 this was the case for 20 of 21 residues across all heterologous viruses and relevant selected sites. The one exception was for site 440_gp120_ and recognition of 25710, where the plasma acquired neutralization activity against 25710 at week 20 even though mutations at site 440_gp120_ were not detected until week 24.

In many cases, the mutant residue sampled by the quasispecies matched that carried by the heterologous virus. For example, we observed D325N_gp120_, H330Y_gp120_, and O363K_gp120_ substitutions in the quasispecies, which match the corresponding amino acid residues in T250, before plasma neutralization activity against T250 emerged. However, while T250 harbors a proline at position 440_gp120_, the quasispecies in animal V634 only sampled an arginine at this position. Indeed, while the heterologous viruses exhibit high variability at position 440_gp120_ **(Fig. 6D)**, none of them carry an arginine. Nevertheless, Q440R_gp120_ was by far the most common escape mutation observed in the quasispecies of SHIV.5MUT-infected macaques that developed bNAbs, suggesting that the loss of the TF glutamine was more important than accommodation of a particular residue at this site. Similarly, position 363_gp120_ is a PNGS in SHIV.5MUT but is highly variable in the heterologous virus panel. The loss of this N-glycan in the quasispecies, which can be achieved by mutation to any non-asparagine residue at 363_gp120_, is likely more important than the particular residue sampled.

Given that increased V1h length and number of N-glycans were also enriched in the quasispecies of SHIV.5MUT-infected macaques that developed bNAbs, we queried their contribution to the acquisition of plasma neutralization breadth in each macaque. V1h elongation was considered a key relevant feature for recognition of six of eight heterologous viruses that had longer V1h regions than SHIV.5MUT (>13 AA) **(Fig. 6D)**. In addition, all eight heterologous viruses had more V1h N-glycans than SHIV.5MUT (>1 N-glycan) **(Fig. 6D)**, and so an increased number of V1h PNGSs was considered a relevant key feature for all viruses. In our analyses, we thus assumed that any increase in these parameters selected for antibodies capable of tolerating longer, more-glycosylated V1h regions. Indeed, the number of V1h N-glycans began to increase early in most SHIV.5MUT-infected macaques, and variants with >1 PNGS were observed in the quasispecies of animal V634 by week 4 post-infection **(Fig. S21)**. V1h elongation was observed in the quasispecies at week 32, contemporaneous with plasma neutralization of 2/6 of the heterologous viruses for which V1h length is relevant (CAP256SU and BJOX02000). While there was low-titer neutralization of the other 4 viruses (T250, Ce1176, TRO11, and CH119) prior to this timepoint, we observed a substantial increase in potency upon emergence of the V1-elongated variants, suggesting that these variants may have selected for bNAb lineage members with enhanced affinity.

Overall, these data provide evidence that viral acquisition of key resistance mutations in response to a developing bNAb lineage *in vivo* provides critical exposure to viral variants that positively select for antibodies within the lineage, which in turn can tolerate diversity at these sites. However, although required, viral acquisition of key resistance mutations was not always sufficient for breadth development. Indeed, there were several instances in which a macaque did not gain plasma neutralization activity against a certain heterologous virus despite the quasispecies having sampled mutations at all key sites (e.g. animal V640, **fig. S31)**. Nonetheless, our analysis of Env-bNAb coevolution in SHIV.5MUT-infected macaques revealed common mechanisms of priming and maturing a diverse set of V3-glycan bNAb lineages.

**Fig. S1. RC1 and 11MUTB immunization induces robust autologous neutralizing responses. (A)** mRNA constructs used in this study. Amino acid sequences are aligned to the BG505 wildtype Env sequence (dots indicate identity and dashes indicate deletions). Stabilization, glycan filling, and germline-targeting mutations are indicated. The RC1 and 11MUTB mRNA constructs were used as immunogens in this study **(Fig. 1A)**. **(B)** Antigenicity of all mRNA-expressed, stabilized, membrane-bound Envs shown in **(A)**. mRNA was transfected into 293F cells and expressed Envs were tested by flow cytometric analysis for binding to a panel of bNAbs (top) as well as conformation-sensitive non-neutralizing antibodies (nnAb, bottom). Binding was quantified as fold-change in mean fluorescence intensity (MFI) over mock-transfected cells, and data points with a >3-fold increase are colored as indicated. **(C)** Plasma neutralization of the indicated virus at 8 and 16 weeks post RC1 immunization, represented as log(1/ID_50_) (left) or reciprocal ID_50_ (right). Protein was given as bolus or escalating dose (ED). Kruskal-Wallis test with Dunn’s correction for multiple comparisons. Not significant (ns) P > 0.05; *P < 0.05. **(D)** Fold reduction in plasma neutralization titer (1/ID_50_) of the indicated alanine point mutant compared to an 11MUTB virus. Open circles represent individual animals and bars represent the median. A horizontal dotted line indicates a threshold of 5-fold reduction. **(E)** Antigenicity of SHIV.5MUT and SHIV.BG505.N332. Neutralization sensitivity was tested using a panel of conformation-sensitive non-neutralizing antibodies (nnAb) and broadly neutralizing antibodies (bNAb). Titers are expressed as IC_50_ in µg/mL. **(F)** Longitudinal viral load in macaques infected with SHIV.5MUT (Groups 1-3) or SHIV.BG505.N332 (Group 4), represented as vRNA copies/mL. Circles indicate animals that were CD8-depleted 2-3 days prior to infection, and triangles indicate animals that were not. Grey lines indicate animals that were excluded from subsequent analysis due to rapid progression to AIDS or abnormally low viral load. **(G)** Neutralization activity of plasma from animal V630 at week 12 post-infection (week 28 post-prime) against the indicated viruses, represented as reciprocal ID_50_. **(H)** Average viral loads in SHIV.5MUT-infected macaques (Groups 1-3) versus SHIV.BG505.N332-infected macaques (Group 4) between weeks 12-48 post-infection, quantified as log(mean viral load). Mann-Whitney U test was used. Not significant (ns). WPI, weeks post-infection.

**Fig. S2. bNAb development in SHIV.5MUT-infected macaques is associated with higher viral loads and autologous neutralization titers. (A)** Average viral loads in SHIV.5MUT-infected macaques (Groups 1-3) between weeks 12-48 post-infection, quantified as log(mean viral load). **(B)** Average autologous neutralization titers in SHIV.5MUT-infected macaques (Groups 1-3), represented as mean(log(IC_50_)). Mann-Whitney U test was used. *P < 0.05; ***P < 0.001.

**Fig. S3. Isolation of bNAb candidates from SHIV.5MUT-infected macaques. (A)** Representative gating strategy for the fluorescence-activated cell sorting used to isolate Env-binding B cells. Live CD3^-^CD8α^-^CD14^-^CD16^-^CD20^+^IgG^+^Env^++^ B cells were sorted using Env trimer probes. **(B)** Gating strategy for the isolation of bone marrow plasma cells. Live CD3^-^CD8α^-^CD14^-^CD16^-^CD20^-^CD31^+^CD102^+^ cells were sorted. WPI, weeks post-infection.

**Fig. S4. Clade-specific breadth of SHIV.5MUT-elicited V3-glycan bNAbs.** **(A)** Summary bar graph highlighting the fraction of heterologous viruses in each clade that is neutralized by the indicated antibody. CRF07 and CRF08 are clade C in Env, and CRF02 is clade A in Env, and hence these CRFs are grouped with the parental clades. **(B)** Sequence diversity at major V3-glycan bNAb epitope signature sites shown for six M-group clades (20). Logos were produced using the AnalyzeAlign tool on the Los Alamos HIV Database ([www.hiv.lanl.gov](http://www.hiv.lanl.gov)) using the database alignment of M-group Envs.

**Fig. S5. Representatives of three AM12 V3-glycan bNAb sub-lineages exhibit diversity in epitope recognition.** **(A)** Epitope mapping of representative members of three AM12 V3-glycan bNAb sub-lineages. Neutralization activity against a panel of Q23 and CAP256SU mutant viruses with mutations at key residues in the V3-glycan epitope is shown, with titers expressed as IC_50_ in µg/mL. **(B)** Distribution of CDRH3 lengths in naïve IgM^+^ peripheral B cells in humans (top) and macaques (bottom), identified by bulk BCR repertoire sequencing. Sequences with CDRH3 ≥20 residues are highlighted. The human dataset was taken from ref (42), and sequences from outlier participant 1070 were excluded. The rhesus dataset was generated in this study and includes 2,172,207 IgM sequences from eight macaques.

**Fig. S6. Evolution of the AM12-352 and AJ09-83 bNAb lineages.** Phylogenetic trees of heavy and light chain sequences derived from next-generation sequencing and isolated mAbs are shown for two rhesus bNAb lineages whose UCAs did not bind to common V1 escape mutants **(Fig. 5F)**. Ancestors were inferred at each node by analyzing longitudinal bulk BCR sequencing data using the SONAR computational analysis pipeline. Sequences are color coded according to their week of isolation and the position of mature bNAbs on the trees are shown. Branches in the heavy chain tree on which amino acid insertions occurred are indicated in red, with four particular CDRH2 insertions highlighted. The bold black line highlights intermediary nodes to the mature bNAb. The tree was constructed from heavy and light chain nucleotide sequences using IgPhyML and the scale bar indicates the number of estimated mutations per site.

**Fig. S7. (con’t on next page)**

**Fig. S7.** **Single-particle cryo-EM workflow. Schematics illustrating the cryo-EM data-processing workflows for all Fab/5MUT and Fab/del4 complex structures, as well as the apo reconstructions of 5MUT, del4, and del8. Representative micrographs, 2D class averages, data processing pipeline, gold-standard Fourier shell correlation (GSFSC) curves for the final reconstructions, and local resolution maps are shown. Azimuthal particle distribution plots indicate modest preferred orientations.**

**Fig. S8. Structural characterization of AM12-352 lineage members. (A)** Top, interfaces of the predicted interactions between the indicated V3-glycan bNAbs and the ^324^GDIR_327_ motif as in **Fig. 2B**. Middle, similar view as top panel showing V3-glycan bNAbs and Env built into cryo-EM density maps. Bottom, same as middle panel but rotated ~90^o^. **(B)** Cryo-EM maps of AM12-340 (left), AM12-347 (middle), and AM12-351 (right) bNAbs in complex with the 5MUT-3fill SOSIP. The heavy chain of each bNAb is shown in a darker shade, and the light chain is shown in a lighter shade. The N332_gp120_ glycan is modeled as cyan spheres and the conserved ^324^GDIR_327_ peptide motif is red. **(C)** Top, surface representations of the V3-glycan bNAbs from **(B)** bound to gp120, with the N332_gp120_ glycan and GDIR motif as depicted in **(B)**. Bottom, interfaces of the predicted interactions between the V3-glycan bNAbs and the GDIR motif. **(D)** Structural overlay of AM12 lineage members demonstrating nearly identical approach angles for binding to Env.

**Fig. S9.** **SHIV.5MUT-elicited bNAbs exhibit diverse angles of approach.** **(A)** Definitions of antibody (Ab) approach angles as described in ref (30). The points shown are the centers of mass (COM) of the V3-glycan epitope, of the V_H_ domain, and of the V_L_ domain. **(B)** For the antibodies shown in **Fig. 2D**, the vectors connecting the V3-glycan epitope COM to the V_H_ domain COM are shown on an Env trimer structure (looking down the three-fold axis).

**Fig. S10. Characterization of inferred V3-glycan bNAb UCAs.** Alignments of heavy chain (HC) and light chain (LC) amino acid sequences of five inferred UCAs, the corresponding mature bNAbs, and closest human V_H_/V_K_ alleles are shown. Analysis of the seven other UCAs is shown in **Fig. 3A.** Somatically mutated residues in the mature bNAb sequences are color-coded by estimated mutation probability, as determined by ARMADiLLO (48).

**Fig. S11. SHIV.5MUT-elicited bNAbs share the majority of their contact residues with the closest human V allele.** The number of contact residues that are (yellow) or are not (blue) identical in the closest human V_H_ and V_K_ alleles are shown for the indicated bNAbs.

**Fig. S12. Immune selection in V1 and V3 Env regions in SHIV.5MUT-infected rhesus macaques.** A highlighter plot of longitudinal Env amino acid sequences obtained by single-genome sequencing of plasma viral RNA is shown for each macaque. Sequences are grouped first by macaque (left) and then by timepoint (right). Each row represents a single sequence, depicted as a string of horizontal pixels with each pixel representing a single amino acid in the alignment. Sequences are shown for the entire Env ectodomain, with a schematic map depicted at top. A single alignment was generated from sequences of all SHIV.5MUT-infected macaques at all timepoints, such that the vertical “stripes” indicate mutations at the same site in different Envs. All sequences are aligned to the SHIV.5MUT Env sequence. Residues with substantial selection are indicated, with colors denoting different amino acids. Timepoints are color-coded on the right. Deletions are denoted by black boxes.

**Fig. S13. Hypoglycosylation of position N133_gp120_ in the 5MUT Env trimer.** Mass spectrometry-based site-specific glycan analysis of MD39-stabilized BG505.N332, BG505.N332.N136P, 5MUT, 5MUT.P136N, del4, del8, and 5MUT-3fill SOSIP proteins. Glycan compositions are grouped into their corresponding categories, with complex-type glycans displayed in pink, hybrid in hatched pink, oligomannose in green, and unoccupied in gray. For sites in which the site-specific data was deduced by employing glycosidase digestion by Endo H followed by PNGase F in O18 water (indicated by asterisks), the peptide modifications which correspond to distinct glycan types are grouped into high mannose in green, fucosylated mannose in hatched green, complex in pink, and unoccupied in gray. Glycan sites that could not be determined are denoted as “N.D.”

**Fig. S14. Compositional site-specific glycan analysis of various BG505- and 5MUT-derived trimers.** Quantification of site-specific occupancy and compositional glycan analysis of MD39-stabilized BG505.N332, BG505.N332.N136P, 5MUT, 5MUT.P136N, del4, del8, and 5MUT-3fill SOSIP proteins. The datasets show the glycoforms found at each potential N-linked glycosylation site (PNGS). Data for oligomannose glycans are shaded in green, hybrid-type glycans in orange, fully processed complex-type glycans in magenta, and the absence of a glycan in gray. Oligomannose-type glycans are categorized according to the number of mannose residues present, hybrid-type glycan by the presence/absence of fucose, and complex-type glycans by the number of processed antenna and the presence/absence of fucose. Data that was obtained using Endo H and PNGase F in O18 water cannot be allocated into the above categories and instead are merged to cover all oligomannose/hybrid compositions or complex-type glycans.

**Fig. S15. Comparative structural analysis of SHIV.5MUT-induced V1- and V3-directed neutralizing antibodies.** Cryo-EM maps of two SHIV.5MUT-induced neutralizing antibodies (NN39-25 and NN39-171) in complex with 5MUT-3fill SOSIP, overlaid with a crystal structure of the human V3-glycan bNAb 10-1074 in complex with BG505.N332 SOSIP (PDB ID: 5T3Z). The heavy chain of each antibody is shown in a darker shade, while the light chain is shown in a lighter shade. The N332_gp120_ glycan is shown in cyan, the conserved ^324^GDIR_327_ peptide motif in red, and the V1 loop (including the N133_gp120_ glycan) in dark blue. NN39-25 exhibits strong V1 loop dependence, whereas NN39-171 and 10-1074 bNAbs predominantly interact with the V3 loop. Contact residues are shown in the table, with red font indicating the four residues differentiating 5MUT from BG505.N332.

**Fig. S16. 5MUT, del4, and del8 trimers exhibit minimal V1 glycosylation.** Density maps showing minimal to no glycosylation within the V1 loops of 5MUT-3fill (top), del4-3fill (middle), and del8-3fill (bottom) SOSIPs.

**Fig. S17. Structural evolution of the V634-136 lineage. (A)** Structural overlay of V634-136 UCA and mature bNAb reveals nearly identical CDRH3 architectures. **(B)** Somatic hypermutations (SHMs) acquired by V634-136-UCA are mapped onto the coordinates. Green spheres represent SHM-derived residues on V634-136-UCA that form contacts with gp120, while blue spheres represent SHM-derived residues outside of the gp120 binding interface. SHMs, somatic hypermutations**.**

**Fig. S18. Early intermediate antibodies in the AM12-352 and AJ09-83 lineages bind common SHIV.5MUT escape mutants that have shortened V1 loops. (A)** Amino acid alignment of inferred unmutated common ancestor (UCA) and inferred ancestors (iA) in the AJ09-83 and AM12-352 lineages. Inferred ancestors for the heavy and light chains of the AJ09-83 lineage are shown, while only inferred ancestors for the heavy chain of the AM12-352 lineage are shown. **(B)** Representative heavy and light chain sequences were selected for synthesis and artificially paired. AJ09-83 intermediate heavy chains were paired with intermediate light chains from a similar developmental stage. AM12-352 intermediate heavy chains were paired with the mature AM12-352 light chain. mRNA-encoded Env trimers were expressed on the surface of 293F cells and binding to UCA and intermediate ancestor mAbs was assessed by flow cytometry. Binding is quantified as fold-change in mean fluorescence intensity (MFI) over mock-transfected cells, and data points with a >3-fold increase are colored as indicated.

**Fig. S19. Env sequence evolution in SHIV-infected rhesus macaques. (A)** Analysis of the evolving Env quasispecies in all SHIV-infected macaques for sites under immune selection. Three groups, including 14 SHIV.5MUT-infected animals that developed bNAbs, 8 SHIV.5MUT-infected animals that did not develop bNAbs, and 14 SHIV.BG505.N332-infected animals that did not develop bNAbs, were subjected to LASSIE analysis, which identifies residues where mutations have altered the encoded amino acid in at least 80% of sequences at one or more timepoints (64). All LASSIE-selected sites are highlighted in blue, with the number indicating the maximum percentage of sequences from any timepoint that exhibit loss of the transmitted founder (TF) residue for each macaque. **(B)** Structural mapping of LASSIE-selected sites in each group onto the BG505 Env (PDB ID: 9EHL). Sites are color-coded as indicated based on recurrence in multiple macaques. The green ellipse demarcates the approximate location of the V3-glycan bNAb epitope. **(C)** Same analysis as shown in **Fig. 6C**, but for sites that were not temporally associated with the acquisition of plasma breadth. Top: Longitudinal mutation frequency (%TF loss) at four sites enriched in SHIV.5MUT-infected macaques that developed bNAbs. Red and black curves denote SHIV.5MUT-infected macaques that did versus did not develop bNAbs, respectively, while grey curves indicate SHIV.BG505.N332-infected animals (all of which failed to develop bNAbs). Thin lines indicate data from individual animals and thick lines represent group averages. Bottom: Mutation frequency (%TF loss) in macaques that developed bNAbs, with the timepoint at which plasma breadth is first detected (defined as at least 1 heterologous virus with reciprocal ID_50_ > 20) set to zero weeks (wks). Different colors indicate different macaques. TF, transmitted founder.

**Fig. S20. Env evolution and development of plasma breadth in rhesus macaque AM12.** Longitudinal patterns of Env mutations and development of plasma neutralization breadth. See **Methods** for a description of the analysis. **(A)** Left: Mutations in the quasispecies of animal AM12 are depicted as logos, where the height of the letter is proportional to its frequency in the Env sequences from the corresponding timepoint. Cyan colored “O” indicates asparagine (N) embedded in an N-linked glycosylation motif (NXS or NXT, where X can be any amino acid except proline). Numbers at the bottom indicate residue positions (HXB2 numbering). Amino acids that match the infecting SHIV.5MUT strain (designated transmitted founder or TF) are hidden. Env positions shown include residues 295-336, which comprise the entire V3 loop and adjacent PNGS, as well as all sites that were deemed to be under selection in the evolving quasispecies by meeting a pre-determined ≥80% TF loss criteria at any timepoint within the first year of infection, as defined by LASSIE (64). Red dots indicate sites under selection that are enriched in animals that developed bNAbs. Middle: Bar plots illustrating the fraction of sequences at each timepoint with a given hypervariable V1 (V1h) loop length or number of glycans. The SHIV.5MUT TF has a V1h length of 13 AA and one V1h glycan, and an increase in either is associated with neutralization resistance. Right: Plasma neutralization over time against eight heterologous viruses, expressed as reciprocal ID_50_. Bottom: See **Fig. 6E** for details regarding figure layout. **(B)** Integrated information from the three graphs in **(A)**. 18/18 amino acid changes, 6/6 V1h elongations, and 8/8 V1h N-glycan additions relevant to a given pseudovirus were detected prior to or concurrent with neutralization breadth being observed in the plasma.

**Fig. S21. Env evolution and development of plasma breadth in rhesus macaque V634.** See **Fig. S20** and **Fig. 6E** for details regarding figure layout and **Methods** for a description of the analysis. **(A)** Longitudinal patterns of mutations in Env and development of neutralization breadth. **(B)** Integrated information from the three graphs in **(A)**. 20/21 amino acid changes in key sites, 4/6 V1h elongations, and 8/8 V1h N-glycan additions relevant to a given pseudovirus were detected prior to neutralization breadth being observed in the plasma.

**Fig. S22. Env evolution and development of plasma breadth in rhesus macaque V635.** See **Fig. S20** and **Fig. 6E** for details regarding figure layout and **Methods** for a description of the analysis. **(A)** Longitudinal patterns of mutations in Env and development of neutralization breadth. **(B)** Integrated information from the three graphs in **(A)**. 11/11 amino acid changes in key sites, 6/6 V1h elongations, and 8/8 V1h N-glycan additions relevant to a given pseudovirus were detected prior to neutralization breadth being observed in the plasma.

**Fig. S23. Env evolution and development of plasma breadth in rhesus macaque V633.** See **Fig. S20** and **Fig. 6E** for details regarding figure layout and **Methods** for a description of the analysis. **(A)** Longitudinal patterns of mutations in Env and development of neutralization breadth. **(B)** Integrated information from the three graphs in **(A)**. 13/13 amino acid changes in key sites, 6/6 V1h elongations, and 8/8 V1h N-glycan additions relevant to a given pseudovirus were detected prior to or concurrent with neutralization breadth being observed in the plasma. The D325N_gp120_ and H330Y_gp120_ variants were rare but present.

**Fig. S24. Env evolution and development of plasma breadth in rhesus macaque V645.** See **Fig. S20** and **Fig. 6E** for details regarding figure layout and **Methods** for a description of the analysis. **(A)** Longitudinal patterns of mutations in Env and development of neutralization breadth. **(B)** Integrated information from the three graphs in **(A)**. 10/10 amino acid changes in key sites, 6/6 V1h elongations, and 8/8 V1h N-glycan additions relevant to a given pseudovirus were detected prior to or concurrent with neutralization breadth being observed in the plasma. O363S_gp120_ was rare at week 8 but common by week 16. Increases in V1h length were initially rare at week 16 but became common by week 32, perhaps contributing to bNAb selection for breadth and potency. Plasma neutralization of Q23 was detected first, perhaps in part because it has a short V1h region and a glycan at O363_gp120_, much like 5MUT.

**Fig. S25. Env evolution and development of plasma breadth in rhesus macaque NN39.** See **Fig. S20** and **Fig. 6E** for details regarding figure layout and **Methods** for a description of the analysis. **(A)** Longitudinal patterns of mutations in Env and development of neutralization breadth. **(B)** Integrated information from the three graphs in **(A)**. 17/17 amino acid changes, 6/6 V1h elongations, and 8/8 V1h N-glycan additions relevant to a given pseudovirus were detected prior to or concurrent with neutralization breadth being observed in the plasma. To recognize 25710 and TRO11, emergence of the E87G_gp120_ variant may be required, rather than simply the loss of the 5MUT-encoded E87_gp120_ residue. Similarly, V1 elongation started at week 8 but was rare until week 28, which coincides with the timepoint at which neutralization potency increased substantially against heterologous viruses with longer V1h loops. Plasma neutralization of Q23 was detected earlier at week 20, perhaps in part because it has a short V1h region much like 5MUT.

**Fig. S26. Env evolution and development of plasma breadth in rhesus macaque V641.** See **Fig. S20** and **Fig. 6E** for details regarding figure layout and **Methods** for a description of the analysis. **(A)** Longitudinal patterns of mutations in Env and development of neutralization breadth. **(B)** Integrated information from the three graphs in **(A)**. 19/21 amino acid changes, 5/5 V1h elongations, and 7/7 V1h N-glycan additions relevant to a given pseudovirus were detected prior to or concurrent with neutralization breadth being observed in the plasma. The two exceptions were D325N_gp120_ and H330Y_gp120_, which were first detected at week 32 despite T250 being weakly neutralized by the plasma at week 24. This potency increased by over 10-fold following subsequent emergence of the D325N_gp120_ and H330Y_gp120_ variants. For this animal, the specific residue at position 87 may be important for recognizing certain viruses, rather than simply the loss of the 5MUT-encoded E87_gp120_ residue. Indeed, E87K/D/G_gp120_ variants were sampled in the quasispecies.

**Fig. S27. Env evolution and development of plasma breadth in rhesus macaque V650.** See **Fig. S20** and **Fig. 6E** for details regarding figure layout and **Methods** for a description of the analysis. **(A)** Longitudinal patterns of mutations in Env and development of neutralization breadth. **(B)** Integrated information from the three graphs in **(A)**. 18/18 amino acid changes, 5/5 V1h elongations, and 6/6 V1h N-glycan additions relevant to a given pseudovirus were detected prior to or concurrent with neutralization breadth being observed in the plasma. O363S_gp120_ is very rare at week 8 but becomes common by week 20. V1h-elongated variants are common by week 32, prior to recognition of heterologous viruses with longer V1h lengths.

**Fig. S28. Env evolution and development of plasma breadth in rhesus macaque AJ09.** See **Fig. S20** and **Fig. 6E** for details regarding figure layout and **Methods** for a description of the analysis. **(A)** Longitudinal patterns of mutations in Env and development of neutralization breadth. **(B)** Integrated information from the three graphs in **(A)**. 6/6 amino acid changes, 2/3 V1h elongations, and 5/5 V1h N-glycan additions relevant to a given pseudovirus were detected prior to or concurrent with neutralization breadth being observed in the plasma. CAP256SU, which has a long V1h region, was weakly neutralized prior to detection of V1h-elongated variants in the quasispecies. The rapid near-complete loss of the critical N332_gp120_ glycan (O332) at week 32 may drive near-complete escape of the maturing bNAb lineage and contribute to the limited breadth and potency observed in the plasma.

**Fig. S29. Env evolution and development of plasma breadth in rhesus macaque NN58.** See **Fig. S20** and **Fig. 6E** for details regarding figure layout and **Methods** for a description of the analysis. **(A)** Longitudinal patterns of mutations in Env and development of neutralization breadth. **(B)** Integrated information from the three graphs in **(A)**. 12/12 amino acid changes, 3/3 V1h elongations, and 5/5 V1h N-glycan additions relevant to a given pseudovirus were detected prior to or concurrent with neutralization breadth being observed in the plasma.

**Fig. S30. Env evolution and development of plasma breadth in rhesus macaque V639.** See **Fig. S20** and **Fig. 6E** for details regarding figure layout and **Methods** for a description of the analysis. **(A)** Longitudinal patterns of mutations in Env and development of neutralization breadth. **(B)** Integrated information from the three graphs in **(A)**. 4/4 amino acid changes, 2/2 V1h elongations, and 3/3 V1h N-glycan additions relevant to a given pseudovirus were detected prior to or concurrent with neutralization breadth being observed in the plasma.

**Fig. S31. Env evolution and development of plasma breadth in rhesus macaque V640.** See **Fig. S20** and **Fig. 6E** for details regarding figure layout and **Methods** for a description of the analysis. **(A)** Longitudinal patterns of mutations in Env and development of neutralization breadth. **(B)** Integrated information from the three graphs in **(A)**. 7/7 amino acid changes, 2/2 V1h elongations, and 3/3 V1h N-glycan additions relevant to a given pseudovirus were detected prior to or concurrent with neutralization breadth being observed in the plasma.

**Fig. S32. Env evolution and development of plasma breadth in rhesus macaque AH82.** See **Fig. S20** and **Fig. 6E** for details regarding figure layout and **Methods** for a description of the analysis. **(A)** Longitudinal patterns of mutations in Env and development of neutralization breadth. **(B)** Integrated information from the three graphs in **(A)**. 6/7 amino acid changes and 3/3 V1h N-glycan additions relevant to a given pseudovirus were detected prior to or concurrent with neutralization breadth being observed in the plasma. O363S_gp120_ was very rare at week 8 but became common by week 24. Addition of a second V1h glycan was first observed at week 12, but this variant was very rare until week 32, coincident with detection of plasma neutralization of T250 and CAP256SU, both of which have two V1h N-glycans. V1h elongation was not observed within the first 48 weeks of infection in this animal and thus was not included in this analysis. V1h length could be contributing to the limited breadth and potency observed in this animal, although other factors may also play a role. Notably, 25710 has a short V1h loop but is not recognized by the plasma.

**Fig. S33. Env evolution and development of plasma breadth in rhesus macaque CM03.** See **Fig. S20** and **Fig. 6E** for details regarding figure layout and **Methods** for a description of the analysis. **(A)** Longitudinal patterns of mutations in Env and development of neutralization breadth. **(B)** Integrated information from the three graphs in **(A)**. 3/4 amino acid changes, 1/2 V1h elongations, and 3/3 V1h N-glycan additions relevant to a given pseudovirus were detected prior to or concurrent with neutralization breadth being observed in the plasma. TRO11 was neutralized at week 20 even though the R308H_gp120_ variant was not observed until week 40 and elongated V1 variants were not observed until week 28. While R308H_gp120_ seemed to be a resistance mutation for most other bNAb lineages, neither AH82 nor CM03 showed much selection involving this residue during the first year of infection, suggesting that this site might not be relevant to the bNAb lineages in these two animals. V1-elongated variants were detected concurrently with plasma neutralization of CAP256SU.

**Fig. S34. Quasispecies sampling of M-group diversity at key sites precedes or coincides with the acquisition of plasma neutralization breadth.** Summary of the analysis in **Fig. 6E** applied to all 14 macaques that developed bNAbs. Mutations are colored as in **Fig. 6E** and are listed in the following format: SHIV.5MUT TF amino acid / position / amino acid(s) sampled by the quasispecies in the indicated macaque. For each animal, the number of times that mutations emerged at the relevant sites prior to or concurrent with the acquisition of plasma neutralization activity against the corresponding virus were counted. Similarly, instances in which the quasispecies sampled increased V1h length or number of PNGS prior to or concurrent with acquisition of neutralization activity against the relevant virus were counted. The few instances in which mutations or V1 features were detected after acquisition of neutralization activity are highlighted in green. Refer to **figs. S20-33** for an in-depth analysis of each animal.

**Data S1. Rhesus macaque demographic information.** Sex, age, infection route, inoculum dosage, and CD8 pre-treatment information for all macaques in this study.

**Data S2.** **SHIV.5MUT-infected rhesus macaques develop heterologous plasma neutralization breadth by week 20-32 post-infection.** Longitudinal plasma neutralization against the indicated viruses, expressed as reciprocal ID_50_.

**Data S3.** **SHIV.BG505.N332-infected rhesus macaques fail to develop plasma neutralization breadth by week 48 post-infection.** Longitudinal plasma neutralization against the indicated viruses, expressed as reciprocal ID_50_.

**Data S4.** **Neutralization screening of representative monoclonal antibodies.** Monoclonal antibodies were synthesized and screened for neutralization activity against the autologous (SHIV.5MUT) and heterologous (T250, CAP256SU, Q23, and Ce1176) viruses. Titers are expressed as IC_50_ in µg/mL. The timepoint and Env probes used to isolate each B cell are listed. Within each animal, antibody names are color-coded by lineage.

**Data S5. Breadth and potency of SHIV.5MUT-elicited V3-glycan bNAbs.** Neutralization breadth and potency of the SHIV.5MUT-elicited V3-glycan bNAbs was assessed using a panel of 130 tier-2 viruses consisting of a 119-virus global panel (blue) plus 11 additional viruses (purple). Viruses are separated by subtype, and sequence features including V1 length, N332/N334_gp120_ glycan status, and V3 epitope sequence are shown. Neutralization titers are expressed as IC_50_ in µg/mL.

**Data S6. Cryo-EM data collection and processing parameters.**

**Data S7. Human V3-glycan bNAb UCA sequences.** Heavy and light chain amino acid sequences of the three human V3-glycan bNAb UCA/iGL inferences used in this study.
